# Intrathecal idursulfase-IT in patients with neuronopathic mucopolysaccharidosis II: Results from a phase 2/3 randomized study

**DOI:** 10.1016/j.ymgme.2022.07.017

**Published:** 2022-08-02

**Authors:** Joseph Muenzer, Barbara K. Burton, Paul Harmatz, Luis González Gutiérrez-Solana, Matilde Ruiz-Garcia, Simon A. Jones, Nathalie Guffon, Michal Inbar-Feigenberg, Drago Bratkovic, Michael Hale, Yuna Wu, Karen S. Yee, David A.H. Whiteman, David Alexanderian

**Affiliations:** a University of North Carolina at Chapel Hill, Chapel Hill, NC, USA; b Ann & Robert H. Lurie Children’s Hospital of Chicago, Northwestern University, Chicago, IL, USA; c UCSF Benioff Children’s Hospital Oakland, Oakland, CA, USA; d Infant Jesus Children’s Hospital, Madrid, Spain; e National Institute of Pediatrics, Mexico City, Mexico; f St Mary’s Hospital, Manchester University NHS Foundation Trust, University of Manchester, Manchester, UK; g Reference Center for Inherited Metabolic Diseases, Hospices Civils de Lyon, Lyon, France; h University of Toronto, Toronto, ON, Canada; i The Hospital for Sick Children, Toronto, ON, Canada; j Women’s and Children’s Hospital, North Adelaide, SA, Australia; k Takeda Development Center Americas, Inc., Cambridge, MA, USA; l Hale Scientific Statistics, LLC, Beaverton, OR, USA; m Takeda Development Center Americas, Inc., Lexington, MA, USA; n Affinia Therapeutics, Inc., Waltham, MA, USA

**Keywords:** Mucopolysaccharidosis II, MPS II, Cognitive impairment, Idursulfase, Intrathecal

## Abstract

Two-thirds of patients with mucopolysaccharidosis II (MPS II; Hunter syndrome) have cognitive impairment. This phase 2/3, randomized, controlled, open-label, multicenter study (NCT02055118) investigated the effects of intrathecally administered idursulfase-IT on cognitive function in patients with MPS II. Children older than 3 years with MPS II and mild-to-moderate cognitive impairment (assessed by Differential Ability Scales-II [DAS-II], General Conceptual Ability [GCA] score) who had tolerated intravenous idursulfase for at least 4 months were randomly assigned (2:1) to monthly idursulfase-IT 10 mg (*n* = 34) via an intrathecal drug delivery device (IDDD; or by lumbar puncture) or no idursulfase-IT treatment (*n* = 15) for 52 weeks. All patients continued to receive weekly intravenous idursulfase 0.5 mg/kg as standard of care. Of 49 randomized patients, 47 completed the study (two patients receiving idursulfase-IT discontinued). The primary endpoint (change from baseline in DAS-II GCA score at week 52 in a linear mixed-effects model for repeated measures analysis) was not met: although there was a smaller decrease in DAS-II GCA scores with idursulfase-IT than with no idursulfase-IT at week 52, this was not significant (least-squares mean treatment difference [95% confidence interval], 3.0 [−7.3, 13.3]; *p* = 0.5669). Changes from baseline in Vineland Adaptive Behavioral Scales-II Adaptive Behavior Composite scores at week 52 (key secondary endpoint) were similar in the idursulfase-IT (*n* = 31) and no idursulfase-IT (*n* = 14) groups. There were trends towards a potential positive effect of idursulfase-IT across DAS-II composite, cluster, and subtest scores, notably in patients younger than 6 years at baseline. In a post hoc analysis, there was a significant (*p* = 0.0174), clinically meaningful difference in change from baseline in DAS-II GCA scores at week 52 with idursulfase-IT (*n* = 13) versus no idursulfase-IT (*n* = 6) among those younger than 6 years with missense iduronate-2-sulfatase gene variants. Overall, idursulfase-IT reduced cerebrospinal glycosaminoglycan levels from baseline by 72.0% at week 52. Idursulfase-IT was generally well tolerated. These data suggest potential benefits of idursulfase-IT in the treatment of cognitive impairment in some patients with neuronopathic MPS II. After many years of extensive review and regulatory discussions, the data were found to be insufficient to meet the evidentiary standard to support regulatory filings.

## 1. Introduction

Mucopolysaccharidosis II (MPS II; Hunter syndrome; OMIM309900) is a rare, X-linked lysosomal storage disease caused by deficient activity of the enzyme iduronate-2-sulfatase (I2S), owing to deletion or pathological variants of the I2S gene (*IDS*) [1–3]. The resulting accumulation of glycosaminoglycans (GAGs) in lysosomes throughout the body leads to a wide range of progressive somatic manifestations [1,2,4–6]. Approximately two-thirds of patients have neuronopathic disease, presenting with cognitive impairment in addition to the somatic manifestations [7]. The age of onset and the rate of disease progression vary considerably among patients. In those with neuronopathic disease, signs and symptoms typically develop between 2 and 4 years of age and often progress rapidly, with death usually occurring in the second decade of life; individuals with non-neuronopathic disease experience progressive somatic signs and symptoms and may survive into the fifth or sixth decade of life [1,2,8].

More than 670 *IDS* variants have been reported, with missense variants being the most common [9–12]. Large deletions/insertions, complex rearrangements, and nonsense and splicing variants are the *IDS* variants most associated with cognitive impairment [9,13,14]. For missense variants, there are multiple reports of associations with both non-neuronopathic (attenuated) and neuronopathic (severe) disease [10,14,15].

Disease-specific treatment for MPS II has been available since 2005 in the form of intravenous (IV) enzyme replacement therapy with idursulfase (Elaprase^®^, Takeda Pharmaceuticals USA, Inc., Lexington, MA, USA). The favorable safety and efficacy profiles of IV idursulfase in improving somatic manifestations of MPS II have been demonstrated in clinical trials [16,17], real-world studies [18,19], and in a statistical modeling study [20]. However, IV idursulfase is not able to cross the blood–brain barrier at therapeutic concentrations and has not been shown to have direct effects on cognitive decline.

A formulation of idursulfase was developed for intrathecal administration (idursulfase-IT). In animal models, administration of idursulfase-IT resulted in effective delivery of the enzyme to the brain and spinal cord with widespread deposition in lysosomes of neurons and oligodendrocytes [21]. Furthermore, in animals deficient in I2S, idursulfase-IT reduced the storage of GAGs in brain tissue and improved morphological features [21]. A phase 1/2 trial (HGT-HIT-045; NCT00920647) investigated the safety of idursulfase-IT administered via an intrathecal drug delivery device (IDDD), in combination with weekly IV idursulfase, in 16 male patients with MPS II and cognitive impairment [22]. Idursulfase-IT was generally well tolerated, and no serious adverse events were considered related to idursulfase-IT. Moreover, after 6 months of treatment with idursulfase-IT 10 mg or 30 mg, mean GAG levels in cerebrospinal fluid (CSF) were reduced by approximately 90%, with most of the reduction apparent after the first dose.

The present phase 2/3 randomized controlled study (HGT-HIT-094; NCT02055118) evaluated the effects of monthly idursulfase-IT in addition to weekly IV idursulfase on cognitive function in children with MPS II and early cognitive impairment, over 52 weeks. The safety profile of idursulfase-IT was also assessed.

## 2. Materials and methods

### 2.1. Participants

Male patients aged > 3 to 18 years with a diagnosis of MPS II and evidence of MPS II-related cognitive impairment were eligible for inclusion in the study. Patients had to have a deficiency in I2S activity of ≤10% of the lower limit of normal as measured in plasma, fibroblasts, or leukocytes (based on the reference laboratory’s normal range), together with either a documented *IDS* variant that leaves the fragile X mental retardation genes (*FMR1*, *FMR2*) intact, or a normal enzyme activity level of one of the other sulfatases as measured in plasma, fibroblasts, or leukocytes (based on the reference laboratory’s normal range). Different criteria for demonstration of cognitive impairment were applied depending on the patient’s age at informed consent (Supplementary Table 1). All patients had to have received and tolerated at least 4 months of treatment with IV idursulfase in the period immediately before screening. Patients were excluded if they had an opening CSF pressure of >30.0 cm H_2_O upon lumbar puncture or had a functioning CSF shunt device.

Eligible patients were enrolled between March 24, 2014, and September 29, 2016, across nine study sites in seven countries (Australia, Canada, France, Mexico, Spain, the UK, and the USA). The last study visit was on September 28, 2017.

### 2.2. Study design

This was a phase 2/3, controlled, randomized, two-arm, open-label, assessor-blinded, multicenter, 52-week study. Eligible patients were randomly assigned (2:1) to receive either idursulfase-IT 10 mg monthly (every 28 days) or no idursulfase-IT treatment; all patients continued to receive IV idursulfase 0.5 mg/kg once a week as standard of care. For children receiving idursulfase-IT, the IV infusion of idursulfase was scheduled at least 48 h after idursulfase-IT administration. Randomization was stratified according to baseline Differential Ability Scales-II (DAS-II) General Conceptual Ability (GCA) [23] scores (≤ 70 or > 70; representing 2 standard deviations [SDs] below the mean [100] and the median point between the upper and lower entry requirements; also, scores ≤70 are defined in the DAS-II manual as indicating low to very low cognitive abilities). The randomization schedule was generated and distributed centrally using an interactive voice response system by Pharmaceutical Product Development, LLC, independent of the sponsor (Supplementary Methods). Participants were assigned a treatment arm based on the next available unassigned random record. The assessors for the primary and secondary endpoints were not informed of the randomization assignments, and families were asked not to share the information with them; assessors were also excluded from the study team and study team meetings.

The study was approved by the relevant institutional review boards/institutional ethics committees and was conducted in compliance with the International Conference on Harmonisation Good Clinical Practice guidelines and the Declaration of Helsinki. For all patients, written informed consent was obtained from the parent(s) or legally authorized guardian(s); assent from the patient was also acquired, if applicable.

A sub-study of the 52-week trial was undertaken in patients younger than 3 years of age at baseline. Patients who completed the 52-week assessment in the phase 2/3 study or sub-study were eligible for enrollment in an open-label, non-randomized extension study to evaluate the long-term safety of idursulfase-IT (SHP609–302; NCT02412787). Results from the ongoing extension study and sub-study are presented elsewhere [24].

### 2.3. Idursulfase-IT and IDDD

Idursulfase-IT is formulated as an isotonic, sterile solution of recombinant human I2S for intrathecal administration. The solution was provided as a 10 mg/mL concentration in preservative-free saline and was intended for single use. Idursulfase-IT was administered via the SOPH-A-PORT^®^ Mini S, Implantable Access Port, Spinal, Mini Unattached, with Guidewire (SOPH-A-PORT Mini S; Sophysa SA, Orsay, France) IDDD. In the phase 1/2 study, idursulfase-IT was administered via the PORT-A-CATH^®^ (Smiths Medical, St Paul, MN, USA) IDDD; however, there were more complications than expected related to the devices, which necessitated surgical revisions and/or removals [22]. Therefore, the SOPH-A-PORT Mini S was used in this study to improve the device performance.

In patients randomly assigned to the idursulfase-IT group, surgical placement of the IDDD was performed, with at least 14 days allowed for recovery before administration of the first dose of idursulfase-IT. Thereafter, patients received idursulfase-IT 10 mg once every 28 days for 52 weeks. Idursulfase-IT could be administered via lumbar puncture if the intrathecal space was inaccessible via the IDDD or in the event of IDDD malfunction; administration via this route was allowed up to 12 times during the study and subject to discussion with the medical monitor. In the no idursulfase-IT group, patients did not undergo surgical placement of the IDDD and did not receive idursulfase-IT.

### 2.4. Endpoints and assessments

The primary endpoint was the change from baseline in cognitive function, assessed by the DAS-II GCA score, at week 52. The DAS-II GCA score is a measure of verbal, non-verbal, and spatial clusters of the DAS-II; therefore, it measures global intellectual ability, with higher values indicating better cognitive function (mean, 100; SD, 15). Changes in the norm-based DAS-II GCA score between two time points provide an assessment of the change in cognitive performance relative to the typical trajectory of change in an age-matched normative sample [23,25]. The DAS-II GCA score was assessed with one of two overlapping, age-based batteries in the DAS-II: the early years battery, designed for children aged 2 years 6 months to 6 years 11 months, and the school age battery, for those aged from 7 years 0 months to 17 years 11 months; these batteries are fully co-normed for ages 5 years 0 months to 8 years 11 months [23]. DAS-II was administered in English, Spanish and (in a translated form) French (i.e. for use in France and Canada).

The key secondary endpoint was the change from baseline in the Vineland Adaptive Behavior-II (VABS-II) Adaptive Behavior Composite (ABC) score at week 52. The VABS-II ABC score provides an overall measure of adaptive behavior ability and is a composite score of domains for communication, daily living, socialization, and motor skills in children (motor skills domain only included for children younger than 7 years; mean, 100; SD, 15) [26].

Patients in both treatment groups were assessed over a total of 13 months (28 days per month) from randomization to the end of study (EOS) evaluation (Supplementary Tables 2 and 3).

Additional secondary endpoints were changes from baseline in the DAS-II GCA and VABS-II ABC scores at weeks 16, 28, and 40, changes from baseline in DAS-II cluster standard scores and subtest T-scores for early years/school age core subtests at weeks 16, 28, 40, and 52, and changes from baseline in VABS-II standard scores of other domains and in V-scale subdomain scores at weeks 16, 28, 40, and 52 (Supplementary Table 4).

Pharmacodynamic endpoints included changes from baseline in total GAG and heparan sulfate (HS) levels in the CSF. Samples of CSF were collected via the IDDD. For patients in the idursulfase-IT group for whom this was not possible and for all patients in the no idursulfase-IT group, CSF samples were obtained by lumbar puncture. Total CSF GAG concentrations were quantified by the study sponsor using a thrombin activity assay [22]. Levels of HS in CSF were determined using a validated liquid chromatography–mass spectrometry/mass spectrometry assay, which measured *N*-butylaniline-derivatized disaccharides from the digestion of CSF HS by heparinases; the lower limit of quantification was 0.100 μM. HS assays were conducted by a sponsor-designated contract research organization (Labcorp Drug Development, Burlington, NC, USA).

Safety assessments comprised adverse events (AEs), clinical laboratory tests (serum chemistry, hematology, urinanalysis), physical and neurological examinations, evaluations of vital signs, 12-lead electrocardiogram (ECG) recordings, and measurements of anti-idursulfase antibodies in the serum and CSF. In the idursulfase-IT group, treatment-emergent AEs were defined as those occurring on or after the date of the first IDDD implantation surgery or of the first dose (whichever was earlier) and at or before the EOS visit (+ 30 days) or 2 weeks after the removal of the last IDDD (whichever was later). In the no idursulfase-IT group, treatment-emergent AEs were defined as those occurring on or after the date of randomization and at or before the EOS visit. AEs were coded using the MedDRA dictionary (version 16.1). Assessments of the safety of the IDDD comprised measures of device implantation, device function, device longevity, and AEs associated with the implantation surgery or device. Antibody testing was conducted by a sponsor-designated contract research organization (Pharmaceutical Product Development, LLC, Richmond, VA, USA), using a bridging electrochemiluminescent immunoassay and the Meso Scale Discovery technology platform (Meso Scale Diagnostics, LLC, Rockville, MD, USA), as described previously [22]. For positive samples, neutralizing activity was detected by an I2S enzymatic activity assay using 4-methylumbelliferyl sulfate as substrate.

### 2.5. Statistical analysis

Based on the findings in the phase 1/2 study (NCT00920647), an approximate decline in DAS-II GCA score of 13 points from baseline (screening) at week 52 was assumed for the control (no idursulfase-IT) group. Allowing for the fact that up to 2 months could elapse from screening to the start of treatment with idursulfase-IT, a mean projected treatment difference of 11 points at week 52 was considered to represent a clinically meaningful treatment difference. With a 2:1 allocation ratio, a sample size of 48 patients (32 patients in the idursulfase-IT group and 16 patients in the no idursulfase-IT group) was estimated to yield 80% power to detect this clinically meaningful difference in the primary endpoint. Safety was evaluated in all randomized patients with any post-randomization safety assessments. All statistical analyses were performed using Statistical Analysis System (SAS) software version 9.3 or higher (SAS Institute, Cary, NC, USA).

#### 2.5.1. Prespecified analyses

The primary efficacy analysis population was the intention-to-treat (ITT) population, defined as all randomized patients. For the primary efficacy analysis, a linear mixed-effects model for repeated measures (MMRM) analysis was employed to compare the least-squares mean change from baseline in DAS-II GCA scores in the idursulfase-IT group with that in the no idursulfase-IT group. In the ITT population, the MMRM included fixed categorical effects for treatment, visit week, treatment by visit week interaction, baseline DAS-II GCA category (≤ 70 and > 70), baseline age group (< 6 and ≥ 6 years), treatment by baseline DAS-II GCA category interaction, treatment by baseline age group interaction, interaction between baseline DAS-II GCA category and baseline age group, genotype, and the continuous covariate of baseline DAS-II GCA score. Unstructured within-patient covariance was applied. The statistical test for the primary endpoint was performed using the MMRM-based *t*-test with a two-sided significance level of 5%.

The statistical test for the key secondary endpoint of change in VABS-II ABC score from baseline at week 52 was also performed using the MMRM-based *t*-test with a two-sided significance level of 5%. The same MMRM model as that used for DAS-II GCA scores was applied, except that it included baseline VABS-II ABC score rather than baseline DAS-II GCA score as the continuous covariate. Additional secondary efficacy endpoints of changes from baseline in standard scores of the clusters of DAS-II at each time point and changes from baseline in standard scores of the VABS-II domains at each time point were also assessed using the MMRM analysis, with the baseline score for each measure included as the continuous covariate. Other secondary endpoints were summarized descriptively by treatment group over time.

In subgroup analyses, changes from baseline in the DAS-II GCA and VABS-II ABC scores were assessed using an MMRM in the following subgroups: patients with baseline DAS-II GCA score ≤ 70 or > 70; patients aged <6 or ≥ 6 years at baseline; and patients aged <55 or ≥ 55 months at baseline. The model included fixed categorical effects for treatment, visit week, treatment by visit week interaction, subgroup, treatment by subgroup interaction, subgroup by visit interaction, and the three-way interaction among treatment, visit week and subgroup, and genotype and baseline DAS-II GCA score as a continuous covariate. Subgroup interactions were assessed with a significance level of 10%. In addition, the change from baseline in DAS-II GCA score at week 52 was compared between treatment groups in the subgroup of patients younger than 6 years at baseline using an MMRM analysis. The MMRM was not applied in the subgroup of patients aged 6 years or older at baseline owing to the small population size.

The changes from baseline in DAS-II GCA scores in the overall population and subgroups were also assessed using a prespecified alternative method, a rate of change (weighted slope) analysis. Only patients with data for at least three time points were included in this analysis. In the first stage of this analysis, the rate parameter of DAS-II GCA score change was estimated for each individual: this was the linear regression slope of the DAS-II GCA scores over the study period. The next stage involved statistical inference of the treatment effect using the individual rate parameters and a weighted generalized linear model, with those with good fits receiving larger weights. The weighted slope estimates were compared between treatment groups using a weighted one-way analysis of variance test.

The main MMRM analysis and the DAS-II GCA results with the alternative rate of change (weighted) analysis highlighted trends in DAS-II composite scores and subtest T-scores suggestive of a particular treatment benefit in patients younger than 6 years at baseline. Therefore, this subgroup was selected for the post hoc genotype analysis.

#### 2.5.2. Post hoc analyses

In the post hoc analysis of the primary endpoint, patients younger than 6 years at baseline were categorized by genotype as having either missense or other types of *IDS* variants. Using the MMRM, the least-squares mean change from baseline in DAS-II GCA score in the idursulfase-IT group was compared with that in the no idursulfase-IT cohort of patients younger than 6 years at baseline for the subgroup with missense genotypes and in that with other genotypes. The difference between treatment groups was tested at week 52 using an MMRM-based *t*-test with a two-sided significance level of 5%.

The changes from baseline in total CSF GAG levels (total GAGs and HS) in the idursulfase-IT and no idursulfase-IT treatment groups were also assessed post hoc for different *IDS* genotype categories (missense vs other) in patients younger than 6 years at baseline.

## 3. Results

### 3.1. Study population

**Fig. 1.**
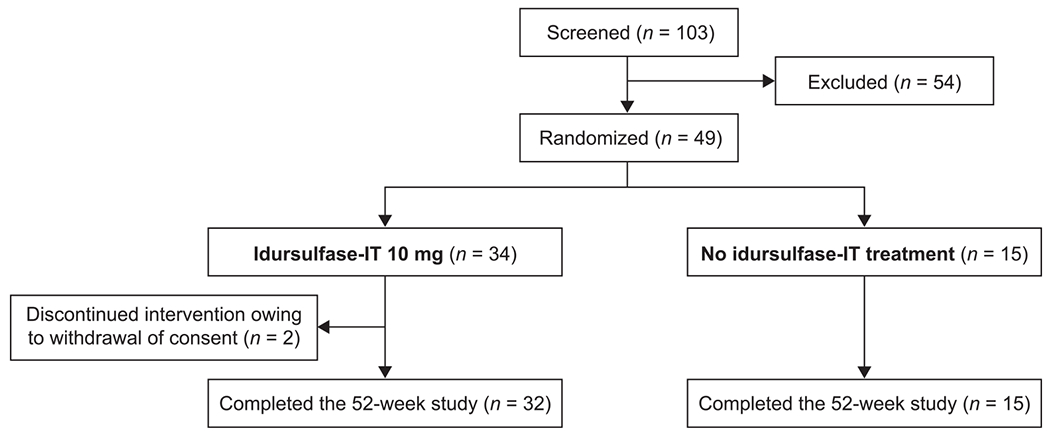
Patient flow for the study. IT, intrathecal.

In total, 49 patients were randomly assigned to receive either idursulfase-IT (*n* = 34) or no idursulfase-IT treatment (*n* = 15; Fig. 1). Two patients in the idursulfase-IT group (6%) discontinued from the study owing to withdrawal of consent; 32 patients in the idursulfase-IT group and all 15 patients in the no idursulfase-IT group completed the study. The median (range) age was 4.63 (3.1–13.0) years (Table 1). For cognitive testing with DAS-II, the early years battery was used for 46 patients and the school age battery was used for five patients (two of whom also contributed some data via the early years battery). In the idursulfase-IT and no idursulfase-IT groups, respectively, 28/34 patients (82%) and 12/15 patients (80%) were younger than 6 years. Missense variants were the most common *IDS* genotype, present in 49.0% of patients overall (50.0% and 46.7% in the idursulfase-IT and no idursulfase-IT groups, respectively). Among those aged <6 years with a missense *IDS* variant, there were nine patients with DAS-II GCA scores ≤70 and 10 with scores >70. Individual patient genotypes are described in Supplementary Table 5.

Overall, 33 patients received at least one dose of idursulfase-IT (Supplementary Table 6). The median (range) number of doses received was 12.0 (2—12) and the median (range) duration of treatment was 9.95 (1.9–10.6) months. In all, 29 patients received at least one dose of idursulfase-IT via IDDD (median [range] number of doses, 12.0 [1–12]) and 15 patients received at least one dose via lumbar puncture (median [range] number of doses, 4.0 [1–12]).

### 3.2. Changes from baseline in DAS-II GCA scores

**Fig. 2.**
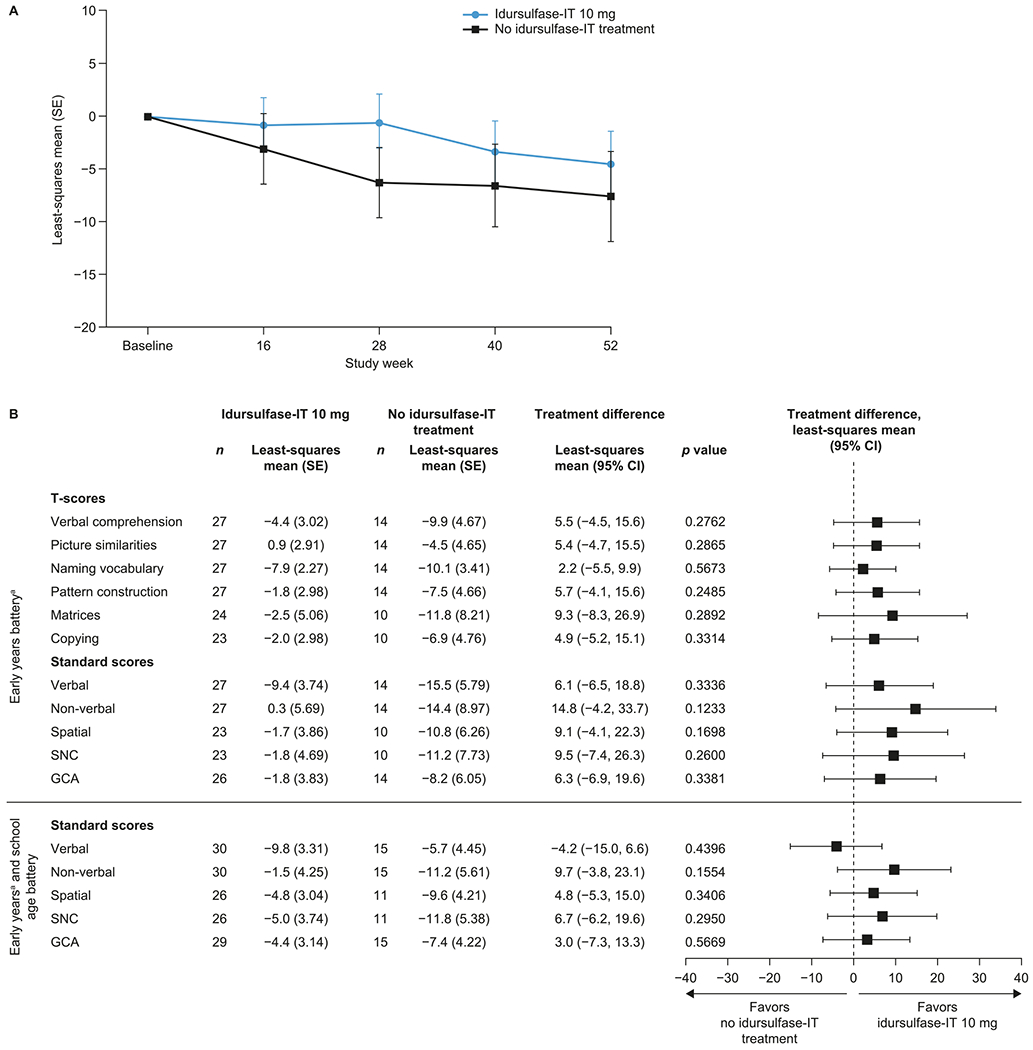
Least-squares mean change from baseline in (A) DAS-II scores over 52 weeks and (B) DAS-II composite, cluster, and subtest scores at week 52 in the ITT population (MMRM analysis). ^a^Subtests included in the early years batteries of the DAS-II differ. In total, 46 of the 49 patients included in the trial were assessed with the early years battery; therefore, these results are presented. T-score data for the school age battery are not available owing to the small number of patients (*n* = 3). CI, confidence interval; DAS-II, Differential Ability Scales-II; GCA, General Conceptual Ability; IT, intrathecal; MMRM, mixed-effects model for repeated measures; SE, standard error; SNC, special non-verbal composite.

In the primary (MMRM) analysis, there was a decrease in DAS-II GCA scores from baseline throughout the study in both treatment groups (Fig. 2A). At week 52, the change in DAS-II GCA score from baseline was smaller in the idursulfase-IT group (*n* = 29) than in the no idursulfase-IT group (*n* = 15), with a least-squares mean treatment difference of 3.0 (95% confidence interval [CI]: −7.3, 13.3). This difference did not reach statistical significance (*p* = 0.5669); thus, the primary endpoint was not met. However, there was a trend towards a positive effect with idursulfase-IT compared with no idursulfase-IT treatment across the DAS-II composite, cluster, and subtest scores (Fig. 2B).

**Fig. 3.**
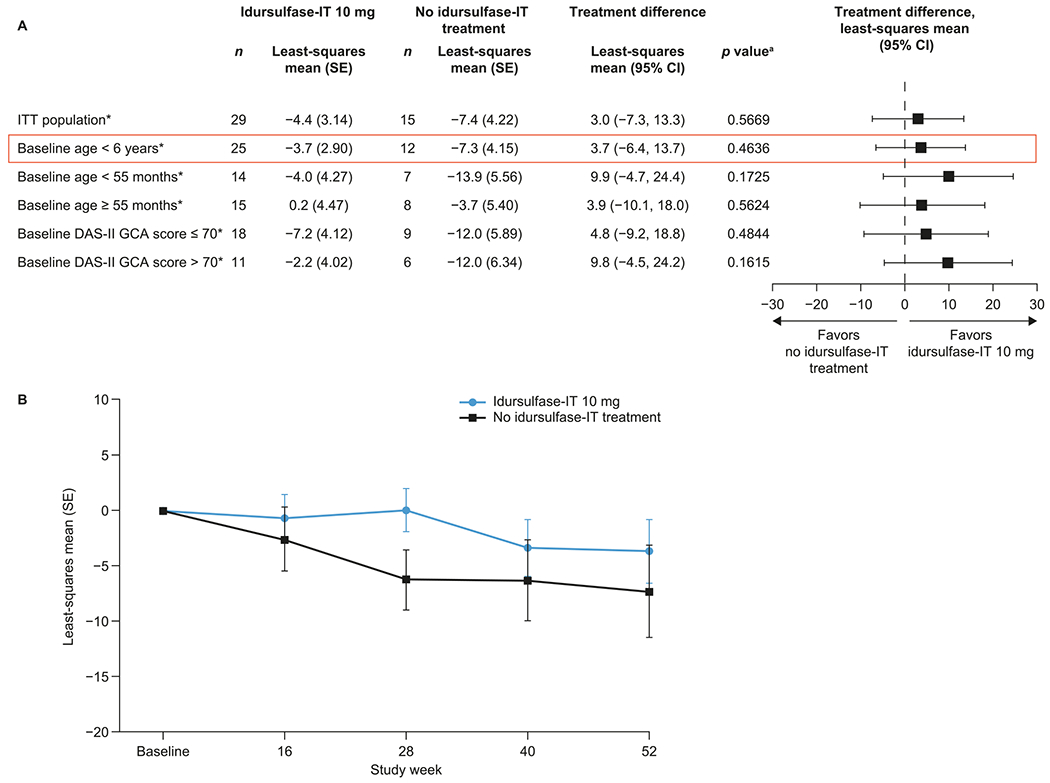
Least-squares mean change from baseline in DAS-II GCA scores over 52 weeks (A) in prespecified subgroups and (B) in patients younger than 6 years at baseline (MMRM analysis). ^a^Based on an MMRM. *n*, number of patients without missing DAS-II GCA scores at week 52. CI, confidence interval; DAS-II, Differential Ability Scales-II; GCA General Conceptual Ability; IT, intrathecal; ITT, intention-to-treat; MMRM, mixed-effects model for repeated measures; SE, standard error.

In the prespecified subgroup MMRM analyses, trends towards potential benefits of idursulfase-IT versus no idursulfase-IT treatment on DAS-II GCA scores were observed across all the subgroups assessed (Fig. 3A). In patients younger than 6 years at baseline, the least-square mean (standard error [SE]) changes from baseline in DAS-II GCA score at week 52 were − 3.7 (2.90) with idursulfase-IT and − 7.3 (4.15) with no idursulfase-IT treatment with a least-squares mean (95% CI) treatment difference of 3.7 (−6.4, 13.7) (Fig. 3A and B). There was also a trend towards a positive effect with idursulfase-IT compared with no idursulfase-IT treatment across DAS-II composite, cluster, and subtest scores, although none of these reached statistical significance (Supplementary Fig. 1).

**Fig. 4.**
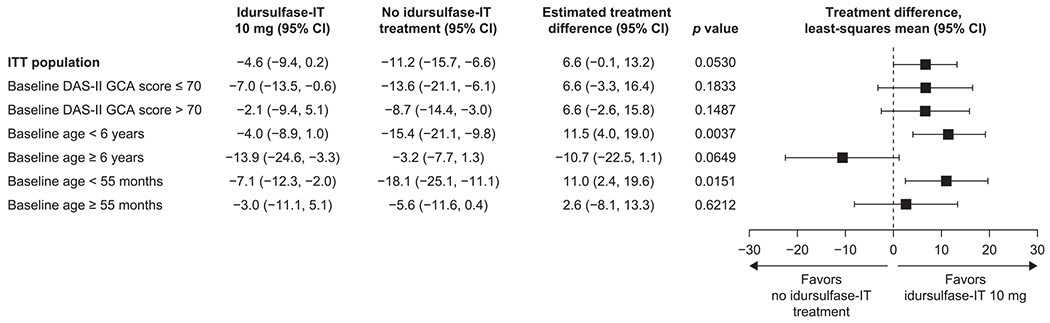
Rate of change (weighted slope) in DAS-II GCA scores by prespecified subgroup. CI, confidence interval; DAS-II, Differential Ability Scales-II; GCA, General Conceptual Ability; IT, intrathecal; ITT, intention-to-treat; SE, standard error.

In the rate of change (weighted slope) analysis of the change from baseline in DAS-II GCA score in the prespecified subgroups, the greatest estimated treatment difference was for patients younger than 6 years at baseline (Fig. 4): in this subgroup, the estimated difference between treatment groups was 11.5 (95% CI: 4.0, 19.0; *p* = 0.0037). A rerandomization test was applied to ensure adequate α control for the weighted rate of change analysis in DAS-II GCA score for patients younger than 6 years at baseline; using 30,000 re-randomizations, the corrected two-tailed *p* value was 0.0454.

### 3.3. Change from baseline in DAS-II GCA score in patients younger than 6 years at baseline by IDS genotype (post hoc analysis)

Among the 40 patients younger than 6 years at baseline, 19 had a missense variant (13 in the idursulfase-IT group and six in the no idursulfase-IT group), five had deletions, seven had frameshift variants, two had nonsense variants, four had intronic variants, one had a splicing variant, and two had an unclassifiable genotype. The median (range) age was 4.4 (3.1–5.7) years in patients with missense variants and 4.1 (3.1–5.9) years in those with other genotypes. Mean (SD) baseline DAS-II GCA score was 69.8 (8.6) in patients with missense *IDS* variants and 67.6 (7.8) in those with other *IDS* variants (Supplementary Table 6). In patients in the idursulfase-IT group younger than 6 years at baseline, exposure to treatment was similar in those with missense variants and those in other genotype categories (Supplementary Table 7).

**Fig. 5.**
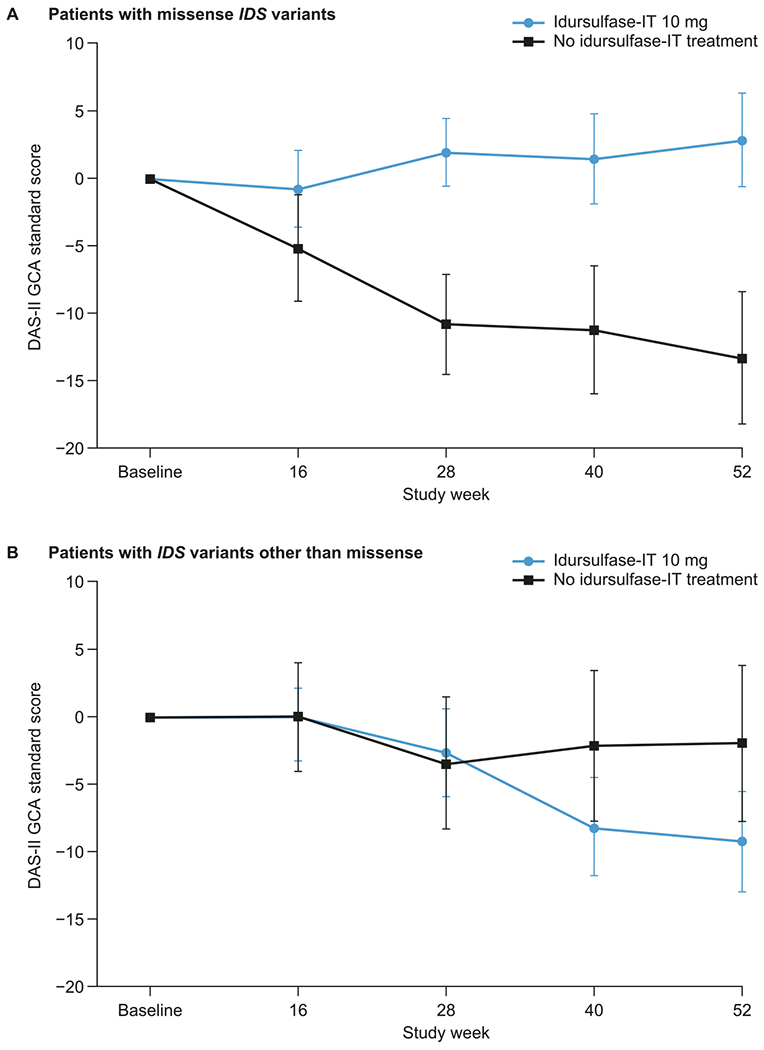
Least-squares mean change (±SE) from baseline in DAS-II GCA scores over 52 weeks in patients younger than 6 years at baseline with (A) missense *IDS* variants and (B) *IDS* variants other than missense (MMRM analysis). DAS-II, Differential Ability Scales-II; *IDS*, iduronate-2-sulfatase gene; IT, intrathecal; GCA, General Conceptual Ability; MMRM, mixed-effects model for repeated measures; SE, standard error.

In patients younger than 6 years at baseline with missense *IDS* variants, there was a significant and clinically meaningful difference in the change from baseline in DAS-II GCA score at week 52 with idursulfase-IT compared with no idursulfase-IT treatment (least-square mean treatment difference, 16.1 [95% CI: 3.3, 28.9; *p* = 0.0174]; Fig. 5). There was also a trend towards a positive effect with idursulfase-IT compared with no idursulfase-IT treatment across the DAS-II composite, cluster, and subtest scores in these patients (Supplementary Fig. 2).

For patients younger than 6 years at baseline with *IDS* variants other than missense, there was no significant difference in the change from baseline in DAS-II GCA score at week 52 between the idursulfase-IT and no idursulfase-IT groups (Fig. 5) and no evidence of a treatment effect across the DAS-II composite, cluster, and subtest scores (Supplementary Fig. 2).

### 3.4. Changes from baseline in VABS-II ABC scores

Changes from baseline in VABS-II ABC scores were similar in the idursulfase-IT and no idursulfase-IT groups at week 52 (least-square mean treatment difference, 0.3 [95% CI: −6.0, 6.6; *p* = 0.9218]) and throughout the study (Supplementary Table 8).

### 3.5. Safety and tolerability

Idursulfase-IT was generally well tolerated throughout the 52-week study (Table 2). There were no deaths, life-threatening AEs, or discontinuations due to AEs. Most AEs were mild or moderate in severity; there were four treatment-emergent severe AEs in three patients in the idursulfase-IT group, including two device catheter kinks or twists and single cases of influenza and acute otitis media. All severe AEs were resolved during the study. No serious AEs were considered related to idursulfase-IT treatment; most were related to the IDDD surgical procedure (29 patients [87.9%] reported at least one related AE), to the IDDD, or to the intrathecal administration process (both reported by 22 patients [66.7%]). In the overall safety population, the incidence of AEs associated with idursulfase-IT administration by lumbar puncture was lower than that for administration via IDDD (27.3% vs 54.5%, respectively); 9 of 15 patients receiving at least one dose via lumbar puncture and 18 of 29 patients receiving at least one dose via IDDD experienced at least one AE associated with the idursulfase-IT administration.

In the idursulfase-IT group, the most common treatment-emergent AEs were vomiting, reported by 75.8% of patients, pyrexia (60.6%), cough (45.5%), and procedural pain (42.4%). In total, 25 patients (75.8%) had an idursulfase-IT-related AE and as noted above, 22 patients (66.7%) had an IDDD-related AE. IDDD failures were reported for four patients (12.1%) and IDDD malfunctions occurred in eight patients (24.2%). In total, 10 patients (30.3%) underwent IDDD-related surgeries in addition to the implantation surgery, with three patients (9.1%) requiring device adjustment; complete device removal without replacement was performed in three patients (9.1%).

The safety profile in the subgroup of patients younger than 6 years with the missense *IDS* genotype was consistent with that observed in the overall safety population (Table 2).

The proportion of patients with normal ECG status at baseline and an abnormal ECG result at any time during the study in the overall safety population, was higher in the idursulfase-IT group than in the no idursulfase-IT group; however, most of the abnormal ECG results were not considered to be clinically meaningful.

### 3.6. Immunogenicity

The numbers of patients who tested positive for serum anti-idursulfase antibodies in the idursulfase-IT and no idursulfase-IT groups, respectively, were 21 (63.6%) and 11 (73.3%) at baseline, and 19 (59.4%) and 11 (73.3%) at week 52. Of these, the numbers of patients who tested positive for serum neutralizing anti-idursulfase-IT antibodies in the idursulfase-IT and no idursulfase-IT groups were 17 (81.0%) and eight (72.7%) at baseline and 12 (63.2%) and four (36.4%) at 52 weeks. Positivity for CSF anti-idursulfase antibodies was recorded in 12 patients (36.4%) in the idursulfase-IT group and seven patients (46.7%) in the no idursulfase-IT group at baseline, and in 10 (32.3%) and seven patients (46.7%) in the idursulfase-IT and no idursulfase-IT groups, respectively, at week 52. For CSF idursulfase neutralizing antibodies, the proportions of patients who tested positive in the idursulfase-IT and no idursulfase-IT groups, respectively, were 16.7% and 0% at baseline, and 10.0% and 14.3%atweek 52. Among those younger than 6 years at baseline, the presence of CSF idursulfase neutralizing antibodies at any time was reported for one of 19 patients (5%) with a missense *IDS* variant (in the no idursulfase-IT group) and for five of 21 patients (29%) with *IDS* variants other than missense (all from the idursulfase-IT group).

### 3.7. Pharmacodynamics

**Fig. 6.**
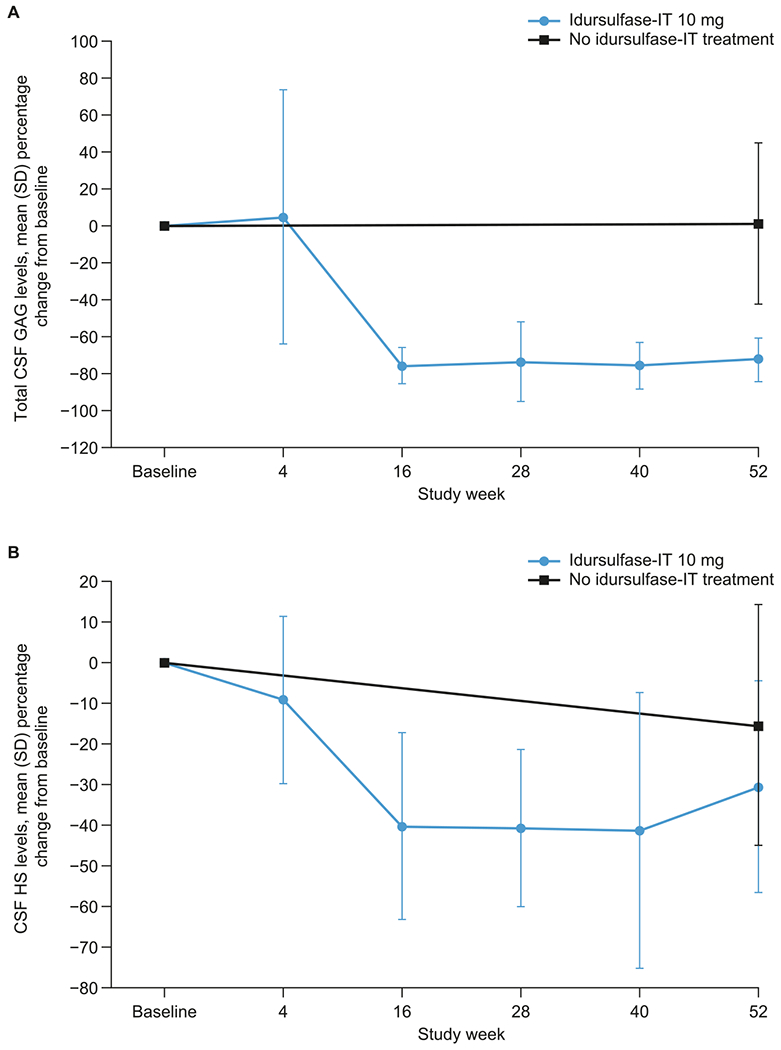
Mean (SD) percentage change from baseline in (A) total CSF GAG levels and (B) CSF HS concentration in the ITT population over 52 weeks. CSF, cerebrospinal fluid; GAG, glycosaminoglycan; HS, heparan sulfate; IT, intrathecal; ITT, intention-to-treat; SD, standard deviation.

In the idursulfase-IT group, the mean (SD) percentage change from baseline in total CSF GAG levels was −72.0% (11.8%) at week 52, with mean reductions from baseline of at least 72% observed from week 16 (Fig. 6A). In the no idursulfase-IT group, changes in total CSF GAG levels over time were minimal, with a mean (SD) percentage change from baseline at week 52 of 1.4% (43.5%).

At baseline, mean (SD) total CSF GAG levels in the idursulfase-IT and no idursulfase-IT groups were similar at 1279.5 (915.2) ng/mL and 1261.5 (811.7) ng/mL respectively but at week 52 these levels were 350.1 (347.3) ng/mL and 1177.9 (667.8) ng/mL; notably, the value for the idursulfase-IT group was closer to the upper limit of normal for healthy individuals, which has been reported to be approximately 200 ng/mL [27].

There was also a reduction from baseline in CSF HS levels with idursulfase-IT (Fig. 6B). At week 52, the mean (SD) percentage change from baseline was −30.6% (26.0%) in the idursulfase-IT group and −15.4% (29.6%) in the no idursulfase-IT group.

There was a rapid and sustained reduction in total CSF GAG levels from baseline with idursulfase-IT in the subgroup of patients younger than 6 years at baseline (Supplementary Fig. 3). Moreover, the reduction in this subgroup was seen both in patients with missense *IDS* genotypes and in those with other *IDS* genotypes (Supplementary Fig. 4). Overall, 35–52% of patients younger than 6 years receiving idursulfase-IT had total CSF GAG levels ≤ 200 ng/mL during weeks 16–52, compared with no patients in the no idursulfase-IT group. The mean (SD) change from baseline in total CSF GAG levels in the subgroup of patients younger than 6 years was −72.4% (12.1%) in the idursulfase-IT group and was essentially unchanged in the no idursulfase-IT group (−0.9% [46.8%]). In those with missense *IDS* variants, there was a mean (SD) percentage change from baseline in total CSF GAG levels of −74.1% (12.0%) at week 52, with 55–75% of patients achieving total CSF GAG levels ≤ 200 ng/mL between weeks 28 and 52. In the no idursulfase-IT group, there was a much smaller mean (SD) percentage change from baseline in total CSF GAG levels (−24.1% [40.1%] at week 52) than in the idursulfase-IT group, and no patients had levels ≤ 200 ng/mL. In patients younger than 6 years at baseline with *IDS* variants other than missense, the proportions of patients achieving total CSF GAG levels of ≤ 200 ng/mL between weeks 28 and 52 were 29–38% and 0% in the idursulfase-IT and the no idursulfase-IT groups, respectively.

There was a reduction from baseline in CSF HS levels with idursulfase-IT in patients younger than 6 years at baseline in both genotype categories during the study, although there was some variation in the results for those with the missense *IDS* genotype (Supplementary Fig. 5). Overall, in patients younger than 6 years at baseline, mean (SD) declines in CSF HS levels were greater in the idursulfase-IT group (−29.9% [26.7%]) than in the no idursulfase-IT group (−19.7% [27.3%]), regardless of genotype.

There were no clear trends in urine GAG concentrations over time and no notable differences between treatment groups in the ITT population (Supplementary Fig. 6).

## 4. Discussion

This phase 2/3 trial investigated the safety and efficacy of idursulfase-IT in a heterogeneous patient population with MPS II and cognitive impairment. The primary endpoint was not met: although the change from baseline in cognitive function (assessed by DAS-II GCA scores) at week 52 was smaller with idursulfase-IT than with no idursulfase-IT, the treatment difference did not reach statistical significance. However, prespecified subgroup analyses using the main MMRM and an alternative rate of change (weighted slope) method, identified a subgroup (patients younger than 6 years at baseline) in which there appeared to be a more pronounced treatment benefit than in the overall population. Subsequently, a post hoc genotype analysis suggested a clinically relevant, significant treatment difference in the change in DAS-II GCA scores from baseline at week 52 for patients younger than 6 years with missense *IDS* variants; no significant treatment difference was observed in the corresponding subgroup of patients with *IDS* variants other than missense. Moreover, at week 52, treatment difference for DAS-II cluster, special non-verbal composite, and GCA standard scores appeared mostly to favor idursulfase-IT treatment over no idursulfase-IT treatment. Similar data were observed for patients who completed the early years battery only.

There was notable variation in the changes from baseline in DAS-II GCA scores between individual patients in both treatment groups, as shown by the size of the SEs of the least-squares means. This probably reflects the heterogeneity of the study patient population, with baseline ages ranging from 3.1 to 13.0 years and baseline DAS-II GCA scores of between 55 and 85. Patient age and disease stage and/or severity of MPS II when idursulfase-IT treatment is initiated may have an impact on outcomes and treatment response over 52 weeks. The heterogeneity of the population may explain, at least in part, the lack of statistical significance for the primary endpoint. Selection of a more tightly defined patient population may have been appropriate; this would have avoided the need to use multiple versions of the DAS-II, thus further minimizing the potential impact of variables on study data.

Subgroup analyses indicated that patients younger than 6 years at baseline may have had a more pronounced treatment response than the overall patient population. A greater treatment response in younger patients is consistent with evidence for the benefits of early treatment of somatic signs and symptoms with IV idursulfase and the biological rationale for early treatment based on the relationship between progressive GAG storage and clinical manifestations in MPS II [28–31]. Moreover, in a post hoc analysis of the subgroup of patients younger than 6 years at baseline with missense *IDS* variants, the least-squares mean (SE) treatment difference for the change from baseline in DAS-II GCA score at week 52 was 16.1 (5.97) points (95% CI: 3.3, 28.9; *p* = 0.0174). Importantly, this was greater than both twice the SE of measurement and 1 SD (15 points) and was therefore considered to be clinically meaningful for standardized scores [32–34]. In contrast, there was no clear difference between treatment groups in patients with other genotypes in the same age group.

Missense variants, the most common *IDS* genotype in patients with MPS II (including among the current study population), lead to enzyme misfolding, catalytic inactivation, premature degradation, or failure of lysosomal targeting [35]. Evidence suggests that single amino acid substitutions in I2S may result in the expression of endogenous proteins with low residual enzymatic activity [36]. This, together with the suggestion that low residual activity may be sufficient to relieve central nervous system symptoms of MPS II [14], may suggest a plausible mechanism for the treatment response observed in patients younger than 6 years at baseline with missense *IDS* variants, namely that administration of idursulfase-IT may supplement the residual enzyme activity enough to reduce lysosomal accumulation of GAGs. This is supported by the greater proportion of patients with reductions of total CSF GAGs to near normal levels with idursulfase-IT among those with missense *IDS* variants than among those with *IDS* variants other than missense [27]. The smaller proportion of patients with CSF anti-idursulfase neutralizing antibodies in the missense *IDS* variant subgroup than in the other variant subgroup (5% vs 29%, respectively) may also have contributed to the different treatment responses. One might speculate that the presence of residual endogenous I2S in patients with missense *IDS* variants (and who therefore have the potential to be positive for cross-reactive immunological material [CRIM]) may reduce the risk of developing anti-idursulfase antibodies with IV idursulfase compared with patients with other pathogenic variants who would have no residual I2S (and who therefore might be expected to be CRIM-negative) [37,38]; however, this has not been assessed in the present study. Development of anti-idursulfase antibodies has been reported after treatment with IV idursulfase and may be associated with a reduced treatment benefit in some patients [39]. Notably, a relationship between immunogenicity and treatment response has also been reported in patients with infantile Pompe disease: a subgroup of these CRIM-negative patients has been shown to develop high titers of neutralizing antibodies and to have worse clinical outcomes during treatment compared with CRIM-positive patients [40,41]. In the overall population in the present study, there was no increase in the proportions of patients with serum or CSF anti-idursulfase antibodies over the analysis period. Another possible factor contributing to the differences in treatment response observed between patients younger than 6 years at baseline with missense *IDS* variants and those with other *IDS* variants is a difference in the rate of progression of disease and, therefore, in the optimal timing of treatment initiation. In a study in patients with neuronopathic MPS II receiving IV enzyme replacement therapy, the rate of decline was slower among patients with missense variants than among those with null-type variants, such as deletions and nonsense variants; however, sample sizes were small, and larger studies are required to establish associations between variants and the anticipated rate of decline [38]. Similarly, a post hoc analysis of data from a 1-year open-label study of 28 patients with MPS II receiving IV idursulfase found associations between safety and efficacy outcomes and genotype, including a more pronounced reduction in liver size among those with missense variants than among those with variants other than missense [39]. Thus, disease progression may not be inherently less severe in patients with missense *IDS* variants than in those with other *IDS* variants, but one possible hypothesis is that it may be more responsive to treatment in the former than the latter group. It is important to remember that missense variants of *IDS* comprise a heterogeneous group from a molecular point of view, with disease severity dependent upon the effect on protein structure. In our study, the evidence for treatment benefit was among those receiving intrathecal therapy compared with those who did not. The possible role of the intrinsic rate of decline and potential for cognitive benefit, related to genotype and age at treatment initiation, remains a topic for further research.

There were no notable differences with idursulfase-IT compared with no idursulfase-IT in changes from baseline in VABS-II ABC score at week 52. It is possible that inclusion of the motor domain in the youngest group may have obscured change. However, in a recent study investigating changes in cognitive function and adaptive behavior in patients with neuronopathic MPS II, VABS-II ABC scores were found to be stable over 2 years even though activities in certain domains would have been expected to decrease significantly in these patients over that time period [42]. The authors noted that it is possible that VABS-II is not sensitive enough to detect small changes in young children with relatively low baseline adaptive behavior and that more patients or a longer study period may be required to see changes in VABS-II ABC scores.

In the idursulfase-IT group, there was a rapid and sustained reduction in total CSF GAG levels from baseline. This treatment response was also observed in the post hoc analysis in patients younger than 6 years at baseline in both genotype categories, with a higher proportion of patients with missense *IDS* variants achieving levels below the upper limit of normal than patients with other *IDS* genotypes. This may be attributable, at least in part, to the smaller proportion of patients with CSF anti-idursulfase neutralizing antibodies among those with missense *IDS* variants than among those with other genotypes and is indicative of a possible greater treatment response in those with missense variants. However, it should be noted that a comprehensive model that fully elucidates the relationship between total CSF GAG levels and cognitive impairment has not yet been developed; GAG accumulation probably has a cumulative effect on disease progression, so reducing total CSF GAG levels from baseline may not be sufficient to stabilize DAS-II GCA scores, particularly in the short term. The reductions in total CSF GAG to normal levels (≤ 200 ng/mL) are more likely to be associated with cognitive benefits.

Idursulfase-IT was generally well tolerated in the study, with no deaths and no discontinuations due to AEs, and no serious AEs that were considered related to idursulfase-IT. The favorable safety profile of idursulfase-IT is consistent with previous results [22]. Overall, the performance of the IDDD was acceptable, although some patients required additional surgical management owing to the complexities of the disease and the device. The alternative option for administration via lumbar puncture ensured patients received uninterrupted treatment. Idursulfase-IT administration via lumbar puncture was also generally well tolerated.

Limitations of this study include the lack of knowledge of the natural history of MPS II when the study was designed and the inability to predict the rate of cognitive decline in this heterogeneous patient population. The duration of the study, 52 weeks, may also not have been long enough to determine the potential benefits of idursulfase-IT treatment. Although patients were required to satisfy strict inclusion criteria, which may limit the generalizability of the results and posed challenges with enrollment, the age range for enrollment was relatively wide, with one patient aged 13 years at baseline. Thus, it is unlikely that our findings were affected considerably by age heterogeneity of enrolled patients, given that the remaining patients were all younger than 8.7 years; although the age range did necessitate the use of two different age-related batteries of the DAS-II. Randomization 1:1 was not considered acceptable to patient groups; therefore, a ratio of 2:1 was selected, limiting the size of the no idursulfase-IT (control) group. Only the assessors of the endpoints were blinded. Single and double blinding of patients and the study investigator were not possible owing to the absence of a sham device, sham injections, or placebo; ethical considerations regarding the conduct of invasive procedures in the absence of any potential treatment benefits precluded having a placebo group.

## 5. Conclusions

Although the primary endpoint of this phase 2/3 study was not met, broader analyses suggest potential trends towards a benefit of idursulfase-IT treatment in stabilizing or slowing the progression of cognitive impairment in some children with neuronopathic MPS II. Notably, a post hoc analysis suggested a statistically significant and clinically relevant benefit of idursulfase-IT on cognitive scores in a subgroup younger than 6 years with missense *IDS* variants, supporting findings from the prespecified exploratory rate of change analysis. Our findings highlight the importance of initiating treatment in younger patients at an earlier stage of disease to improve outcomes. Results from this study also show the potential contribution of genotype and associated propensity for antibody development in the level of treatment response. After many years of extensive review and regulatory discussions, the data were found to be insufficient to meet the evidentiary standard to support regulatory filings. idursulfase-IT will continue to be made available to patients who are currently enrolled in the ongoing open-label extension studies until another approved treatment is available to address the cognitive symptoms.

## Supplementary Material

Supplementary

## Figures and Tables

**Table 1:** Demographics and baseline characteristics (ITT population).

	Idursulfase-IT (*n* = 34)	No idursulfase-IT (*n* = 15)	Overall (*N* = 49)
Age, years			
Mean (SD)	5.0 (1.5)	5.3 (2.6)	5.1 (1.9)
Median (range)	4.6 (3.1, 8.7)	4.8 (3.1, 13.0)	4.6 (3.1, 13.0)
Patients by age group, *n* (%)			
Aged < 6 years	28 (82.4)	12 (80.0)	40 (81.6)
Aged ≥ 6 years	6 (17.6)	3 (20.0)	9 (18.4)
Race, *n* (%)			
White	23 (67.6)	12 (80.0)	35 (71.4)
Asian	4 (11.8)	0	4 (8.2)
Black or African American	1 (2.9)	0	1 (2.0)
Other	6 (17.6)	3 (20.0)	9 (18.4)
Height, cm			
Mean (SD)	111.7 (9.5)	110.9 (11.9)	111.5 (10.2)
Median (range)	109.4 (95.7, 140.0)	107.9 (93.0, 137.7)	108.8 (93.0, 140.0)
Weight, kg			
Mean (SD)	24.5 (4.9)	25.3 (8.4)	24.8 (6.1)
Median (range)	23.8 (18.5, 39.8)	23.3 (17.0, 48.4)	23.6 (17.0, 48.4)
Baseline DAS-II GCA score			
Mean (SD)	68.4 (8.3)	67.3 (7.5)	68.0 (8.0)
Median (range)	67.5 (55, 85)	66.0 (56, 78)	67.0 (55, 85)
Patients by baseline DAS-II			
GCA score category, *n* (%)			
DAS-II GCA score ≤ 70	20 (58.8)	9 (60.0)	29 (59.2)
DAS-II GCA score > 70	14 (41.2)	6 (40.0)	20 (40.8)
Patients by type of *IDS* variant, *n* (%)			
Missense	17 (50.0)	7 (46.7)	24 (49.0)
Frameshift	5 (14.7)	3 (20.0)	8 (16.3)
Large deletion or complete deletion/large rearrangement	5 (14.7)	0 (0)	5 (10.2)
Intronic	2 (5.9)	2 (13.3)	4 (8.2)
Nonsense	3 (8.8)	1 (6.7)	4 (8.2)
Splice site	1 (2.9)	0 (0)	1 (2.0)
Unclassifiable	1 (2.9)	2 (13.3)	3 (6.1)

DAS-II, Differential Ability Scales-II; GCA, General Conceptual Ability; *IDS*, iduronate-2-sulfatase gene; IT, intrathecal; ITT, intention-to-treat; SD, standard deviation.

**Table 2:** Summary of TEAEs^a^ by treatment group at week 52 in the phase 2/3 study (safety population).

	Overall safety population		Patients in the safety population aged <6 years at baseline with missense *IDS* variants	
	Idursulfase-IT (*n* = 33)	No idursulfase-IT (*n* = 15)	Idursulfase-IT (*n* = 12)	No idursulfase-IT (*n* = 6)
At least one AE	33 (100.0)	14 (93.3)	12 (100)	6 (100)
At least one severe AE	3 (9.1)	0	0	0
At least one SAE	12 (36.4)	2 (13.3)	4 (33.3)	1 (16.7)
At least one life-threatening AE	0	0	0	0
Discontinuation due to an AE	0	0	0	0
Deaths	0	0	0	0
At least one AE related to IV idursulfase infusion	8 (24.2)	6 (40.0)	3 (25.0)	3 (50.0)
At least one AE related to idursulfase-IT	25 (75.8)	–	10 (83.3)	–
At least one IDDD surgical procedure related AE	29 (87.9)	–	12 (100)	–
At least one IDDD-related AE	22 (66.7)	–	9 (75.0)	–
At least one IT administration process-related AE	22 (66.7)	–	10 (83.3)	–
Associated with administration via IDDD	18 (54.5)	–	9 (75.0)	–
Associated with administration via lumbar puncture	9 (27.3)	–	5 (41.7)	–
Most common TEAEs by preferred term				
Vomiting	25 (75.8)	3 (20.0)	9 (75.0)	2 (33.3)
Pyrexia	20 (60.6)	7 (46.7)	6 (50.0)	3 (50.0)
Cough	15 (45.5)	4 (26.7)	6 (50.0)	3 (50.0)
Procedural pain	14 (42.4)	–	5 (41.7)	–
Nasopharyngitis	13 (39.4)	5 (33.3)	5 (41.7)	4 (66.7)
Headache	12 (36.4)	1 (6.7)	4 (33.3)	1 (16.7)
Diarrhea	10 (30.3)	2 (13.3)	4 (33.3)	1 (16.7)
Ear infection	9 (27.3)	4 (26.7)	3 (25.0)	1 (16.7)
Heart rate increased	7 (21.2)	4 (26.7)	3 (25.0)	2 (33.3)

Data are shown as number of patients (%) reporting at least one AE.

AE, adverse event; EOS, end of study; IDDD, intrathecal drug delivery device; *IDS*, iduronate-2-sulfatase gene; IT, intrathecal; IV, intravenous; SAE, serious adverse event; TEAE, treatment-emergent AE.

a TEAEs for the no idursulfase-IT group are defined as all AEs occurring on or after the date of randomization and on or before the EOS visit TEAEs for the idursulfase-IT group are defined as all AEs occurring on or after the date of the first IDDD implantation surgery or first dose (whichever is earlier) and on or before the EOS visit (+ 30 days) or 2 weeks after the removal of the last IDDD (whichever is later).

## Data Availability

The datasets, including the redacted study protocol, redacted statistical analysis plan and individual participants’ data supporting the results reported in this article, will be made available within three months from initial request to researchers who provide a methodologically sound proposal. The data will be provided after its de-identification in compliance with applicable privacy laws, data protection and requirements for consent and anonymization.

## Abbreviations:

ABC: Adaptive Behavior Composite
AE: adverse event
CI: confidence interval
CRIM: cross-reactive immunological material
CSF: cerebrospinal fluid
DAS-II: Differential Ability Scales-II
ECG: electrocardiogram
GAG: glycosaminoglycan
GCA: General Conceptual Ability
HS: heparan sulfate
I2S: iduronate-2-sulfatase
IDDD: intrathecal drug delivery device
IDS: iduronate-2-sulfatase gene
IT: intrathecal
IV: intravenous
MMRM: linear mixed-effects model for repeated measures
MPS II: mucopolysaccharidosis II
SD: standard deviation
SE: standard error
VABS-II: Vineland Adaptive Behavior Scales-II
