# Supplementary material for: Intrathecal idursulfase-IT in patients with neuronopathic mucopolysaccharidosis II: Results from a phase 2/3 randomized study
Source: Mol Genet Metab. Author manuscript; Available in PMC 2024 Jan 30. (PMC10826424; doi:10.1016/j.ymgme.2022.07.017)
Supplement: Supplementary [file NIHMS1948757-supplement-Supplementary.docx]

**Supplementary Materials**

**Intrathecal idursulfase-IT in patients with neuronopathic mucopolysaccharidosis II: results from a phase 2/3 randomized study**

Joseph Muenzer, Barbara K. Burton, Paul Harmatz, *et al.*, on behalf of the HGT-HIT-094 study group

**Supplementary methods**

*Randomization*

The randomization list was generated based on a schedule. The randomization record was assigned based on the first available record sorted by sequential record number from the schedule. For central randomization, the participant was assigned a treatment arm based on the next available unassigned random record. The next random record was determined by lowest block and lowest sequence number where the following database fields were null: participant, assigned date, stratum, and site. There were 600 randomization numbers, with 300 assigned to each of the two strata. Within each stratum there were 98 blocks consisting of two blocks of six numbers and 96 blocks of three numbers.

**Supplementary Table 1.** Demonstration of cognitive impairment: requirements for study inclusion by age at the time of informed consent.

|  | Age at study baseline | | |
| --- | --- | --- | --- |
|  | <3 years (sub-study) | ≥3 to <13 years | ≥13 to <18 years |
| DAS-II GCA score 55–85 (inclusive) | – | ✓ | ✓^a^ |
| DAS-II GCA score > 85 | – | ✓^a^ | – |
| BSID-III DQ score 55–85 (inclusive) | ✓ | – | – |

The value 55 represents 3 SDs below the mean (100); the value 85 represents 1 SD below the mean (100).

^a^Only if supported by evidence of a decrease in DAS-II GCA score of ≥ 10 points over 12 months from a previously documented test result during the observational study HGT-HIT-090 (NCT01822184).

BSID-III, Bayley Scales of Infant Development-III; DAS-II, Differential Ability Scales-II; DQ, developmental quotient; GCA, General Conceptual Ability; SD, standard deviation.

**Supplementary Table 2.** Schedule of events and assessments performed in patients in the idursulfase-IT group.

|  | **Weeks −4 to −1** | **Week 0** | **Week 2 (+ 7 days)^a^** | | | **Weeks 4, 8, 12, 16, 20, 24, 28, 32, 36, 40, 44, and 48 of idursulfase-IT dosing (± 7 days)** | | | **Week 52/EOS (± 7 days)** | **7 (± 2) days after EOS** |
| --- | --- | --- | --- | --- | --- | --- | --- | --- | --- | --- |
|  |  |  | **Before surgery** | **Surgery^b^** | **Follow-up** | **Before treatment^c^** | **IT injection** | **Follow-up^d^** |  |  |
| Informed consent^e^ | ✓ |  |  |  |  |  |  |  |  |  |
| Review of study entry criteria | ✓ |  |  |  |  |  |  |  |  |  |
| Medical history | ✓ |  |  |  |  |  |  |  |  |  |
| MPS II diagnosis and genotype | ✓^f^ |  |  |  |  |  |  |  |  |  |
| Echocardiogram^g^ | ✓ |  |  |  |  |  |  |  |  |  |
| Physical and neurological examination | ✓ |  | ✓^h^ |  | ✓^i^ | ✓ |  | ✓ | ✓ |  |
| Height and weight | ✓ |  | ✓^h^ |  |  | ✓^j^ |  |  | ✓ |  |
| Head circumference | ✓ |  |  |  |  | ✓ ^j^ |  |  | ✓ |  |
| Hearing assessment | ✓ |  |  |  |  | ✓^k^ |  |  | ✓ |  |
| Neurodevelopmental assessment | ✓ |  |  |  |  | ✓^k^ |  |  | ✓ |  |
| 12-lead ECG | ✓ |  | ✓^h^ |  |  | ✓ | ✓^j,l^ |  | ✓ |  |
| Vital signs | ✓ |  | ✓^h^ | ✓ |  | ✓ | ✓^m^ |  | ✓ |  |
| Clinical laboratory tests (hematology, serum chemistry, urinanalysis) | ✓ |  | ✓^h^ |  |  | ✓^j^ |  |  | ✓ |  |
| Coagulation tests^n^ |  |  | ✓ |  |  |  |  |  |  |  |
| Urine GAG and creatinine | ✓ |  |  |  |  | ✓^j^ |  |  | ✓ |  |
| Anti-idursulfase antibody | ✓ |  |  |  |  | ✓^j^ |  |  | ✓ |  |
| General anesthesia | ✓ |  |  | ✓ |  |  |  |  | ✓ |  |
| Brain MRI | ✓ |  |  |  |  |  |  |  | ✓ |  |
| ICP measurement (by lumbar puncture) | ✓ |  |  |  |  |  |  |  | ✓ |  |
| CSF sample collection^o^ | ✓^p^ |  |  | ✓ |  |  | ✓^q^ |  | ✓ |  |
| Serum albumin | ✓ |  |  |  |  | ✓^j^ |  |  | ✓ |  |
| Randomization |  | ✓^r^ |  |  |  |  |  |  |  |  |
| IDDD implantation |  |  |  | ✓ |  |  |  |  |  |  |
| X-ray (additional imaging may be required)^s^ |  |  |  | ✓ |  |  |  |  | ✓ |  |
| Idursulfase-IT injection |  |  |  |  |  |  | ✓^t^ |  |  |  |
| Serum sample for PK |  |  |  |  |  |  | ✓^u^ |  |  |  |
| Health status questionnaire | ✓ |  |  |  |  |  |  |  | ✓ |  |
| Concomitant medications, therapies/interventions, medical/surgical procedures | ✓ | ✓ | ✓ | ✓ | ✓ | ✓ | ✓ | ✓ | ✓ | ✓ |
| AEs | ✓ | ✓ | ✓ | ✓ | ✓ | ✓ | ✓ | ✓ | ✓ | ✓ |

^a^Patients in the USA who were randomized in the pivotal study before FDA authorization of investigational use of the SOPH-A-PORT Mini S IDDD and treated with idursulfase-IT via lumbar puncture will have the assessments originally planned at week 2 (+ 7 days) performed at the time of the delayed device implantation surgery.

^b^At least 14 days were allowed for recovery following the placement of the IDDD before the administration of the first dose of idursulfase-IT.

^c^From week 28 onward, pre-treatment assessments could be performed on the same day as administration of idursulfase-IT if the patient could arrive at the study site early in the day and if the investigator deemed this clinically appropriate.

^d^The follow-up visit was to occur the day after idursulfase-IT administration during the first 6 months of idursulfase-IT treatment (i.e. weeks 4–24). For the last 6 months of treatment with idursulfase-IT (i.e. from week 28 onward) and in the absence of any safety concerns, patients could complete the safety follow-up visit on the same day as idursulfase-IT administration before discharge.

^e^Informed consent (and patient assent, if applicable) was obtained from the patient’s parent(s)/legally authorized guardian(s) before beginning screening assessments.

^f^The results of patients’ genotype analyses had to be known before randomization.

^g^This assessment did not need to be performed if an echocardiogram taken in the 3 months before study start was available and deemed satisfactory for evaluation of anesthesia risk.

^h^The assessments indicated did not need to be repeated if completed as part of screening assessments in the 7 days before surgery.

^i^The assessments indicated were performed before discharge. It was expected that, for most patients, post-surgical follow-up would occur in week 2 (i.e. 1–2 days after surgery).

^j^The assessments indicated were performed at 3-month intervals (i.e. at idursulfase-IT dosing weeks 4, 16, 28, and 40).

^k^These neurodevelopmental and hearing assessments were performed at idursulfase-IT dosing weeks 16, 28, and 40 (± 7 days).

^l^The 12-lead ECG was performed in the 4 hours from administration of idursulfase-IT at weeks 4, 16, 28, and 40.

^m^Patients remained under observation in the hospital setting (e.g. infusion center, PACU [recovery suite], observation unit, short-stay center) for 4 hours after idursulfase-IT injection for vital signs and other safety assessments. Vital signs were collected at the following time points (± 10 minutes) in relation to administration of idursulfase-IT: in the 15 minutes before administration, 30 minutes after administration, 60 minutes after administration, 120 minutes after administration, and 4 hours after administration. Thereafter, if the investigator deemed the patient to be clinically stable, the patient could leave the hospital setting (except on visit weeks 4, 24, and 48, because serial blood sampling for PK evaluation was planned for those time points). The patient may have needed to be examined the following day.

^n^Pre-surgery coagulation tests were performed by the local laboratory.

^o^CSF samples were collected via the IDDD or lumbar puncture and were used to analyze standard laboratory parameters (chemistries, cell counts), GAG levels, albumin levels, concentration of idursulfase enzyme, and the presence of anti-idursulfase antibodies. Analyses of CSF samples for antibodies and albumin were performed at idursulfase-IT dosing weeks 4, 16, 28, and 40.

^p^The CSF sample was obtained via lumbar puncture while the patient was under general anesthesia.

^q^The CSF sample was obtained at each idursulfase-IT dosing week, before the injection of idursulfase-IT.

^r^Patients were randomized after they had completed screening assessments and met all eligibility criteria. The day of randomization was day 0.

^s^X-rays could be performed to check placement of the device and as needed throughout the study.

^t^On idursulfase-IT dosing weeks, the IV infusion of idursulfase was scheduled to occur a minimum of 48 hours after administration of idursulfase-IT.

^u^Serum samples for PK analysis were obtained at idursulfase-IT dosing weeks 4, 24, and 48. Samples were collected in the 15 minutes (± 5 minutes) before administration of idursulfase-IT and at 30 minutes (± 5 minutes), 60 minutes (± 5 minutes), 120 minutes (± 5 minutes), 4 hours (± 5 minutes), 6 hours (± 5 minutes), 8 hours (± 15 minutes), 12 hours (± 15 minutes), 24 hours (± 15 minutes), 30 hours (± 15 minutes), 36 hours (± 15 minutes) after the start of idursulfase-IT administration.

AE, adverse event; CSF, cerebrospinal fluid; FDA, US Food and Drug Administration; GAG, glycosaminoglycan; IDDD, intrathecal drug delivery device; ECG, electrocardiogram; EOS, end of study; GAG, glycosaminoglycan; ICP, intracranial pressure; IT, intrathecal; IV, intravenous; MRI, magnetic resonance imaging; PACU, post-anesthesia care unit; PK, pharmacokinetic.

**Supplementary Table 3**. Schedule of events and assessments performed patients in the no idursulfase-IT group.

|  | **Weeks −4 to −1** | **Week 0** | **Week 2 (+ 7 days)** | **Weeks 4, 16, 28, and 40**  **(± 7 days)** | **Weeks 8, 12, 20, 24, 32, 36, 44, and 48 (± 7 days)** | **Week 52/EOS (± 7 days)** | **7 (± 2) days after EOS** |
| --- | --- | --- | --- | --- | --- | --- | --- |
| Informed consent^a^ | ✓ |  |  |  |  |  |  |
| Review of study entry criteria | ✓ |  |  |  |  |  |  |
| Medical history | ✓ |  |  |  |  |  |  |
| MPS II diagnosis and genotype | ✓^b^ |  |  |  |  |  |  |
| Echocardiogram^c^ | ✓ |  |  |  |  |  |  |
| Physical and neurological examination | ✓ |  |  | ✓ |  | ✓ |  |
| Height and weight | ✓ |  |  | ✓ |  | ✓ |  |
| Head circumference | ✓ |  |  | ✓ |  | ✓ |  |
| Hearing assessment | ✓ |  |  | ✓^d^ |  | ✓ |  |
| Neurodevelopmental assessment | ✓ |  |  | ✓^d^ |  | ✓ |  |
| 12-lead ECG | ✓ |  |  | ✓ |  | ✓ |  |
| Vital signs | ✓ |  |  | ✓ |  | ✓ |  |
| Clinical laboratory tests (hematology, serum chemistry, urinanalysis) | ✓ |  |  | ✓ |  | ✓ |  |
| General anesthesia | ✓ |  |  |  |  | ✓ |  |
| Urine GAG and creatinine levels | ✓ |  |  | ✓ |  | ✓ |  |
| Anti-idursulfase antibody testing (serum) | ✓ |  |  | ✓ |  | ✓ |  |
| Anti-idursulfase antibody testing (CSF) | ✓ |  |  |  |  | ✓ |  |
| Brain MRI | ✓ |  |  |  |  | ✓ |  |
| ICP measurement (by lumbar puncture) | ✓ |  |  |  |  | ✓ |  |
| CSF sample collection^e^ | ✓ |  |  |  |  | ✓ |  |
| Serum albumin | ✓ |  |  |  |  | ✓ |  |
| Randomization |  | ✓^f^ |  |  |  |  |  |
| Health status questionnaire | ✓ |  |  |  |  | ✓ |  |
| Concomitant medications, therapies/interventions, medical/surgical procedures | ✓ | ✓ | ✓ | ✓ | ✓ | ✓ | ✓ |
| AEs | ✓ | ✓ | ✓ | ✓ | ✓ | ✓ | ✓ |

^a^Informed consent (and patient assent, if applicable) was obtained from the patient’s parent(s)/legally authorized guardian(s) before beginning screening procedures.

^b^The results of patients’ genotype analyses had to be known before randomization.

^c^This assessment did not need to be performed if an echocardiogram taken within 3 months of study start was available and deemed satisfactory for evaluation of anesthesia risk.

^d^These neurodevelopmental and hearing assessments were performed at weeks 16, 28, and 40 (± 7 days).

^e^The CSF sample was obtained via lumbar puncture while the patient was under general anesthesia. The samples were used to analyze standard laboratory parameters (chemistries, cell counts), GAG levels, albumin levels, concentration of idursulfase enzyme, and presence of anti-idursulfase antibodies.

^f^Patients were randomized after they have completed screening assessments and met all eligibility criteria. The day of randomization is day 0.

AE, adverse event; CSF, cerebrospinal fluid; GAG, glycosaminoglycan; IDDD, intrathecal drug delivery device; ECG, electrocardiogram; EOS, end of study; GAG, glycosaminoglycan; ICP, intracranial pressure; IT, intrathecal; MPS II, mucopolysaccharidosis II; MRI, magnetic resonance imaging; PK, pharmacokinetic.

**Supplementary Table 4**. Clinical trial endpoints.

| **Primary efficacy endpoint** |
| --- |
| - Change from baseline in DAS-II GCA score at week 52 |
| **Key secondary endpoint** |
| - Change from baseline in VABS-II ABC score at week 52 |
| **Additional secondary efficacy endpoints^a^** |
| - Change from baseline in the DAS-II GCA score at weeks 16, 28, and 40 - Change from baseline in the VABS-II ABC score at weeks 16, 28, and 40 - Changes from baseline in DAS-II cluster standard scores at weeks 16, 28, 40, and 52 - Changes from baseline in age-equivalent scores, development quotients, and T-scores in the early years and school age core subtests of the DAS-II at weeks 16, 28, 40, and 52 - Changes from baseline in VABS-II standard scores of other domains at weeks 16, 28, 40, and 52 - Changes from baseline in age-equivalent scores, development quotients, and V-scale scores of sub-domains of the VABS-II at weeks 16, 28, 40, and 52 |
| **Pharmacodynamic endpoints^a^** |
| - Change from baseline in total CSF GAG levels - Changes from baseline in CSF HS levels |
| **Safety assessments** |
| - Treatment-emergent AEs, clinical laboratory test results, physical and neurological examination findings, vital signs, and ECG results - Anti-idursulfase antibody response in serum and CSF during treatment |

^a^Reported in this publication.

ABC, Adaptive Behavior Composite; AE, adverse event; CSF, cerebrospinal fluid; DAS-II, Differential Ability Scales-II; ECG, electrocardiogram; GAG, glycosaminoglycan; GCA, General Conceptual Ability; HS, heparan sulfate; VABS-II, Vineland Adaptive Behavior Scales-II.

**Supplementary Table 5**. Individual patient genotypes.

| **Patient number** | **Treatment group** | **Baseline age, years** | **Genotype** | **Amino acid substitution** | **Variant type** |
| --- | --- | --- | --- | --- | --- |
| **Patients younger than 6 years at baseline with missense *IDS* variants** | | | | | |
| 1 | Idursulfase-IT | 5.7 | c.134A>G | p.D45G | Missense |
| 2 | Idursulfase-IT | 4.6 | c.1403G>A | p.R468Q | Missense |
| 3 | Idursulfase-IT | 4.1 | c.1472C>T | p.S491F | Missense |
| 4 | Idursulfase-IT | 4.8 | c.1400C>T | p.467L | Missense |
| 5 | Idursulfase-IT | 4.4 | c.998C>T | p.S333L | Missense |
| 6 | Idursulfase-IT | 3.9 | c.283A>G | p.R95G | Missense |
| 7^a^ | Idursulfase-IT | 3.3 | c.1403G>C | p.R468P | Missense |
| 8 | Idursulfase-IT | 4.4 | c.257C>T | p.P86L | Missense |
| 9 | Idursulfase-IT | 5.5 | c.1504T>G | NA | Missense |
| 10 | Idursulfase-IT | 4.5 | c.998C>T | NA | Missense |
| 11 | Idursulfase-IT | 3.4 | c.1402C>T | NA | Missense |
| 12 | Idursulfase-IT | 5.0 | c.305T>C | NA | Missense |
| 13 | Idursulfase-IT | 5.1 | c.1402C>T | NA | Missense |
| 14 | No idursulfase-IT | 3.4 | c.1402C>T | p.R468W | Missense |
| 15 | No idursulfase-IT | 4.8 | c.1402C>T | R468W | Missense |
| 16 | No idursulfase-IT | 3.1 | c.685C>T | p.H229Y | Missense |
| 17 | No idursulfase-IT | 4.8 | c.686A>G | p.H229R | Missense |
| 18 | No idursulfase-IT | 3.8 | c.257C>T | NA | Missense |
| 19 | No idursulfase-IT | 3.9 | c.1402C>T | NA | Missense |
| **Patients aged 6 years or older at baseline with missense *IDS* variants** | | | | | |
| 20 | Idursulfase-IT | 6.4 | c.1504T>G | p.W502G | Missense |
| 21 | Idursulfase-IT | 8.6 | c.263G>A | NA | Missense |
| 22 | Idursulfase-IT | 8.7 | c.1402C>T | NA | Missense |
| 23 | Idursulfase-IT | 8.0 | c.239A>G | p.Q80R | Missense |
| 24 | No idursulfase-IT | 7.8 | c.134A>G | p.D45V | Missense |
| **Patients with *IDS* variants other than missense** | | | | | |
| 25 | Idursulfase-IT | 4.1 | c.542insA | p.N181fsX17 | Frameshift |
| 26 | Idursulfase-IT | 4.0 | c.1091delC | p.P364fsX26 | Frameshift |
| 27 | Idursulfase-IT | 3.9 | c.782delC | NA | Frameshift |
| 28 | Idursulfase-IT | 3.6 | c.993delT | p.F331fsX8 | Frameshift |
| 29 | Idursulfase-IT | 3.2 | c.1017delA | NA | Frameshift |
| 30 | No idursulfase-IT | 3.2 | c.1381_1384dupATTG | p.462fsX3 | Frameshift |
| 31 | No idursulfase-IT | 5.5 | c.780_781delCCinsG (exon 6) | NA | Frameshift |
| 32 | No idursulfase-IT | 7.9 | c.1220_1221delTT | – | Frameshift |
| 33 | Idursulfase-IT | 4.0 | Deletion of exons 1–8 | – | Large deletion |
| 34 | Idursulfase-IT | 4.1 | Inversion between intron 7 of *IDS* and a region near exon 3 of *IDS-2* resulting in abnormal juxtaposition of exons 8 and 9 of *IDS* to the *IDS-2* region | – | Complete deletion/ large rearrangement |
| 35 | Idursulfase-IT | 4.7 | NA | – | Complete deletion/ large rearrangement |
| 36 | Idursulfase-IT | 3.9 | Attempts to amplify the coding exons of *IDS* failed | – | Complete deletion/ large rearrangement |
| 37 | Idursulfase-IT | 3.1 | Inversion between intron 7 of *IDS* and a region near exon 3 of *IDS-2* | – | Complete deletion/ large rearrangement |
| 38 | Idursulfase-IT | 4.5 | c.419-2A>G | – | Intronic mutation |
| 39 | Idursulfase-IT | 4.8 | c.879+1G>A | – | Intronic mutation |
| 40 | No idursulfase-IT | 4.9 | c.709-2A>G | – | Intronic mutation |
| 41 | No idursulfase-IT | 3.9 | c.419-6delT | – | Intronic mutation |
| 42 | Idursulfase-IT | 4.8 | c.1093G>T | p.G365X | Nonsense |
| 43 | Idursulfase-IT | 7.6 | c.1186C>T | p.Q396X | Nonsense |
| 44 | Idursulfase-IT | 5.1 | c.577G>T | – | Nonsense |
| 45 | No idursulfase-IT | 13.0 | c.1327C>T | NA | Nonsense |
| 46 | Idursulfase-IT | 4.8 | c.1122C>T | p.G374G | Splice site mutation |
| 47 | Idursulfase-IT | 7.3 | Abnormal *IDS* sequencing analysis in exon 8 | – | Unclassifiable |
| 48 | No idursulfase-IT | 5.9 | Hemizygous pathogenic variant proving MPS II | – | Unclassifiable |
| 49 | No idursulfase-IT | 3.5 | Abnormal *IDS* sequencing analysis. p.128X hemizygous nonsense variant in *IDS* considered to be pathogenic | – | Unclassifiable |

^a^Patient 7 was assigned to the idursulfase-IT group but withdrew from the study before receiving the first dose.

del, deletion; dup, duplication; fs, frameshift; *IDS*, iduronate-2-sulfatase gene; *IDS-2*, second *IDS* locus; ins, insertion; IT, intrathecal; MPS II, mucopolysaccharidosis II; NA, not available.

**Supplementary Table 6.** Demographics and baseline characteristics in patients younger than 6 years at baseline.

|  | **Missense *IDS* variants** | | | ***IDS* variants other than missense^a^** | | |
| --- | --- | --- | --- | --- | --- | --- |
|  | **Idursulfase-IT**  **(*n* = 13)** | **No idursulfase-IT**  **(*n* = 6)** | **Overall**  **(*N* = 19)** | **Idursulfase-IT**  **(*n* = 15)** | **No idursulfase-IT**  **(*n* = 6)** | **Overall**  **(*N* = 21)** |
| Age at randomization, years  Mean (SD)  Median (range) | 4.5 (0.7)  4.5 (3.3, 5.7) | 4.0 (0.7)  3.8 (3.1, 4.8) | 4.3 (0.7)  4.4 (3.1, 5.7) | 4.2 (0.6)  4.1 (3.1, 5.1) | 4.5 (1.1)  4.4 (3.2, 5.9) | 4.3 (0.8)  4.1 (3.1, 5.9) |
| Age at diagnosis, years  Mean (SD)  Median (range) | 1.4 (1.3)  1.3 (0, 4.4) | 2.5 (1.2)  2.6 (0.4, 4.2) | 1.8 (1.4)  1.5 (0, 4.4) | 2.7 (0.8)  2.6 (1.0, 3.8) | 1.7 (1.0)  1.9 (0.6, 3.2) | 2.4 (0.9)  2.4 (0.6, 3.8) |
| Race, *n* (%) |  |  |  |  |  |  |
| White | 8 (61.5) | 5 (83.3) | 13 (68.4) | 11 (73.3) | 4 (66.7) | 15 (71.4) |
| Asian | 2 (15.4) | 0 | 2 (10.5) | 1 (6.7) | 0 | 1 (4.8) |
| Black or African American | 0 | 0 | 0 | 1 (6.7) | 0 | 1 (4.8) |
| Other | 3 (23.1) | 1 (16.7) | 4 (21.1) | 2 (13.3) | 2 (33.3) | 4 (19.0) |
| Height, cm  Mean (SD)  Median (range) | 110.1 (6.0)  108.8 (102.3, 122.1) | 106.7 (6.5)  104.1 (101.0, 118.8) | 109.0 (6.2)  106.9 (101.0, 122.1) | 107.3 (5.6)  106.7 (95.7, 117.9) | 105.4 (6.9)  107.0 (93.0, 112.0) | 106.8 (5.9)  106.7 (93.0, 117.9) |
| Weight, kg  Mean (SD)  Median (range) | 22.3 (2.4)  22.7 (19.1, 25.7) | 22.1 (5.1)  21.7 (17.0, 30.8) | 22.2 (3.3)  22.7 (17.0, 30.8) | 24.0 (3.7)  23.7 (18.3, 26.4) | 22.0 (3.2)  21.9 (18.3, 31.9) | 23.4 (3.6)  23.4 (18.3, 31.9) |
| Baseline DAS-II GCA score  Mean (SD)  Median (range) | 70.0 (9.6)  71.0 (55, 85) | 69.3 (6.5)  70.5 (60, 75) | 69.8 (8.6)  71.0 (55, 85) | 67.6 (7.9)  67.0 (56, 81) | 67.7 (8.2)  66.0 (59, 78) | 67.6 (7.8)  67.0 (56, 81) |
| Patients by baseline DAS-II GCA score category, *n* (%)  ≤ 70  > 70 | 6 (46.2)  7 (53.8) | 3 (50.0)  3 (50.0) | 9 (47.4)  10 (52.6) | 10 (66.7)  5 (33.3) | 4 (66.7)  2 (33.3) | 14 (66.7)  7 (33.3) |

^a^The category ‘*IDS* variants other than missense’ includes nonsense (*n* = 2), frameshift (*n* = 7), deletion (*n* = 5), splice site (*n* = 1), intronic (*n* = 4) and unclassifiable (*n* = 2) variants.

DAS-II, Differential Ability Scales-II; GCA, General Conceptual Ability; *IDS*, iduronate-2-sulfatase gene; IT, intrathecal; SD, standard deviation.

**Supplementary Table 7**. Doses received and period of treatment.

|  | **Idursulfase-IT 10 mg** | | |
| --- | --- | --- | --- |
|  | **Idursulfase-IT treatment**  **(*n* = 34)** | **Patients aged  < 6 years at baseline with missense *IDS* variants**  **(*n* = 13)** | **Patients aged  < 6 years at baseline with *IDS* variants other than missense^a^**  **(*n* = 15)** |
| **IT injections** |  |  |  |
| Patients with at least one idursulfase-IT injection received, *n* | 33 | 12 | 15 |
| Mean (SD) number of idursulfase-IT injections | 11.5 (1.77) | 11.8 (0.39) | 11.7 (0.62) |
| Median (range) number of idursulfase-IT injections | 12.0 (2, 12) | 12.0 (11, 12) | 12.0 (10, 12) |
| **IT injections via IDDD** |  |  |  |
| Patients with at least one idursulfase-IT injection received via IDDD, *n* | 29 | 12 | 12 |
| Mean (SD) idursulfase-IT injections via IDDD | 10.3 (2.96) | 10.1 (3.26) | 11.3 (1.14) |
| Median (range) idursulfase-IT injections via IDDD | 12.0 (1, 12) | 12.0 (1, 12) | 11.5 (8, 12) |
| **IT injections via lumbar puncture** |  |  |  |
| Patients with at least one idursulfase-IT injection received via lumbar puncture, *n* | 15 | 5 | 6 |
| Mean (SD) number of idursulfase-IT injections via lumbar puncture | 5.4 (4.42) | 4.2 (3.96) | 6.7 (5.28) |
| Median (range) number of idursulfase-IT injections via lumbar puncture | 4.0 (1, 12) | 3.0 (1, 11) | 7.0 (1, 12) |
| **Duration of idursulfase-IT treatment, months** |  |  |  |
| Mean (SD) | 9.75 (1.454) | 10.02 (0.167) | 9.94 (0.471) |
| Median (range) | 9.95 (1.9, 10.6) | 9.95 (9.8, 10.3) | 9.95 (9.0, 10.6) |

^a^The category ‘*IDS* variants other than missense’ includes nonsense, frameshift, deletion, splicing, and unclassifiable variants.

IDDD, intrathecal drug delivery device; *IDS*, iduronate-2-sulfatase; IT, intrathecal; ITT, intention-to-treat; SD, standard deviation.

**Supplementary Table 8.** Least-squares mean change from baseline in VABS-II ABC scores (MMRM analysis; ITT population).

|  |  | **Idursulfase-IT 10 mg**  **(*n* = 34)** | **No idursulfase-IT treatment**  **(*n* = 15)** | **Least-squares mean difference** |
| --- | --- | --- | --- | --- |
| Week 16 | *n* | 31 | 15 |  |
|  | Least-squares mean (SE) | −3.1 (1.86) | −2.8 (2.21) | −0.3 (2.72) |
|  | 95% CI | −6.8, 0.7 | −7.3, 1.7 | −5.8, 5.3 |
| Week 28 | *n* | 29 | 15 |  |
|  | Least-squares mean (SE) | −1.8 (2.01) | −1.1 (2.45) | −0.7 (3.01) |
|  | 95% CI | −5.8, 2.3 | −6.0, 3.9 | −6.8, 5.4 |
| Week 40 | *n* | 30 | 14 |  |
|  | Least-squares mean (SE) | −4.7 (1.97) | −3.3 (2.41) | −1.5 (2.95) |
|  | 95% CI | −8.7, −0.8 | −8.1, 1.6 | −7.4, 4.5 |
| Week 52/EOS | *n* | 31 | 14 |  |
|  | Least-squares mean (SE) | −5.0 (2.05) | −5.3 (2.55) | **0.3 (3.11)** |
|  | 95% CI | −9.1, −0.8 | −10.4, −0.1 | **−6.0, 6.6** |
|  | *p* value |  |  | **0.9218** |

ABC, Adaptive Behavior Composite; CI, confidence interval; EOS, end of study; IT, intrathecal; ITT, intention-to-treat; MMRM, mixed-effects model for repeated measures; SE, standard error; VABS-II, Vineland Adaptive Behavior Scales-II.


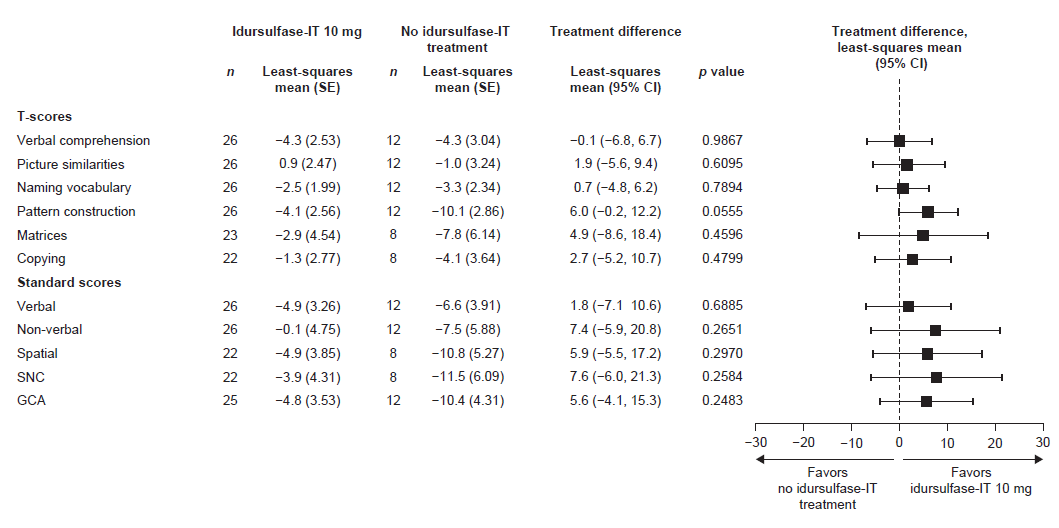


**Supplementary Fig. 1.** DAS-II composite, cluster, and subtest scores at week 52 in patients younger than 6 years at baseline (MMRM analysis; early years data only).

CI, confidence interval; DAS-II, Differential Ability Scales-II; GCA, General Conceptual Ability; IT, intrathecal; MMRM, mixed-effects model for repeated measures; SE, standard error; SNC, special non-verbal composite.


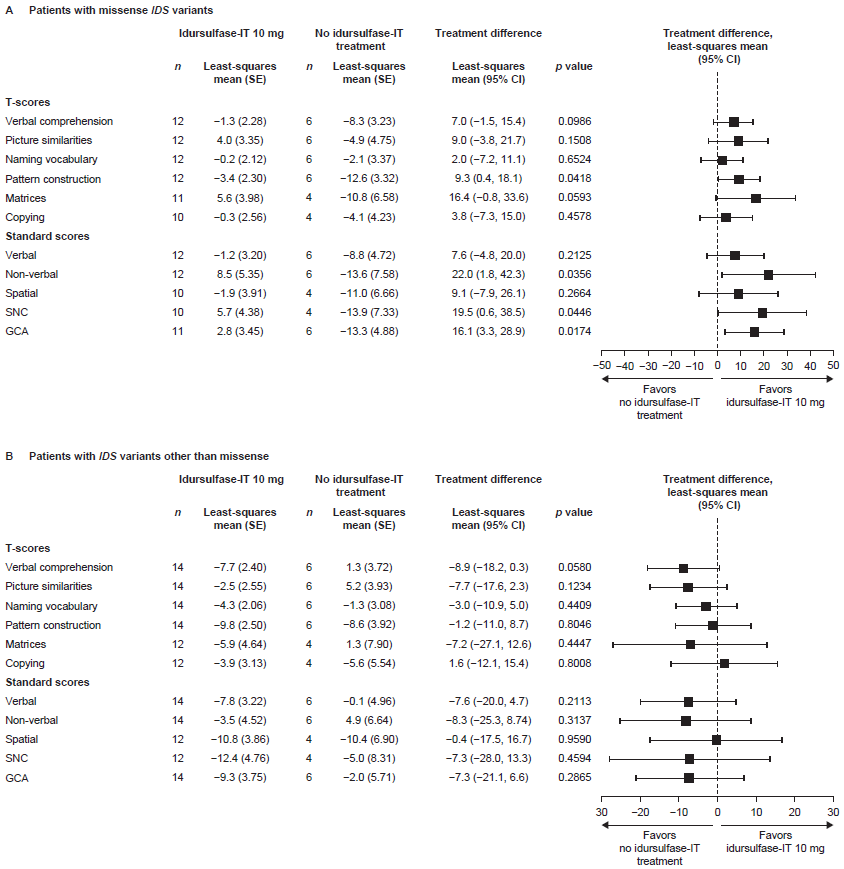


**Supplementary Fig. 2.** DAS-II composite, cluster, and subtest scores at week 52 in patients younger than 6 years at baseline with (A) missense *IDS* variants and (B) *IDS* variants other than missense (MMRM analysis).

CI, confidence interval; DAS-II, Differential Ability Scales-II; GCA, General Conceptual Ability; *IDS*, iduronate-2-sulfatase gene; IT, intrathecal; MMRM, mixed-effects model for repeated measures; SE, standard error; SNC, special non-verbal composite.


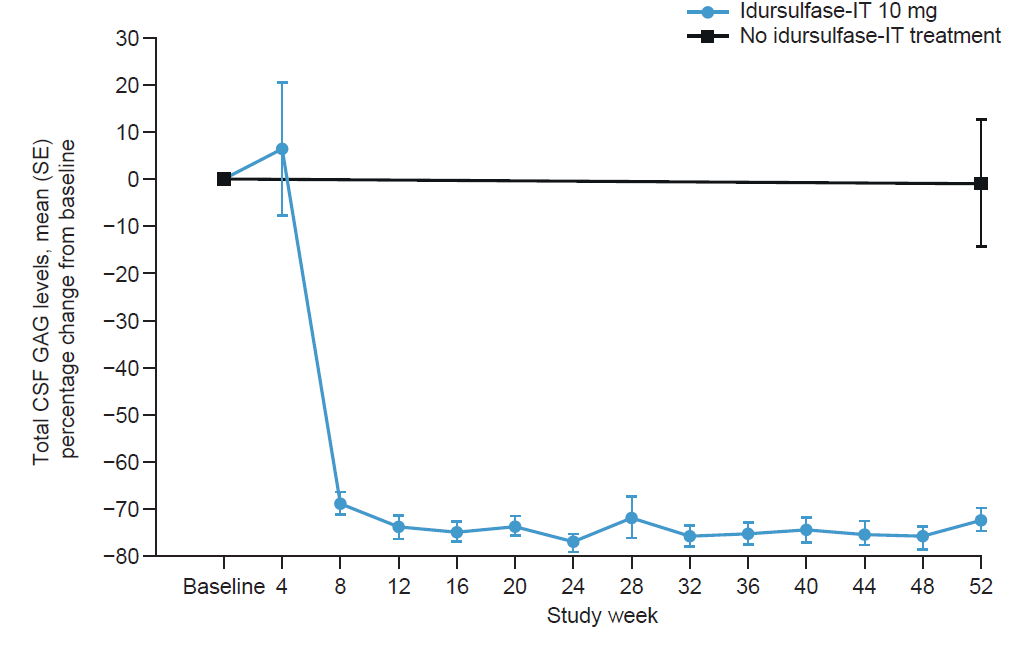


**Supplementary Fig. 3.** Mean percentage change from baseline in total CSF GAG levels over 52 weeks in the overall group of patients younger than 6 years at baseline.

CSF, cerebrospinal fluid; GAG, glycosaminoglycan; IT, intrathecal; SE, standard error.


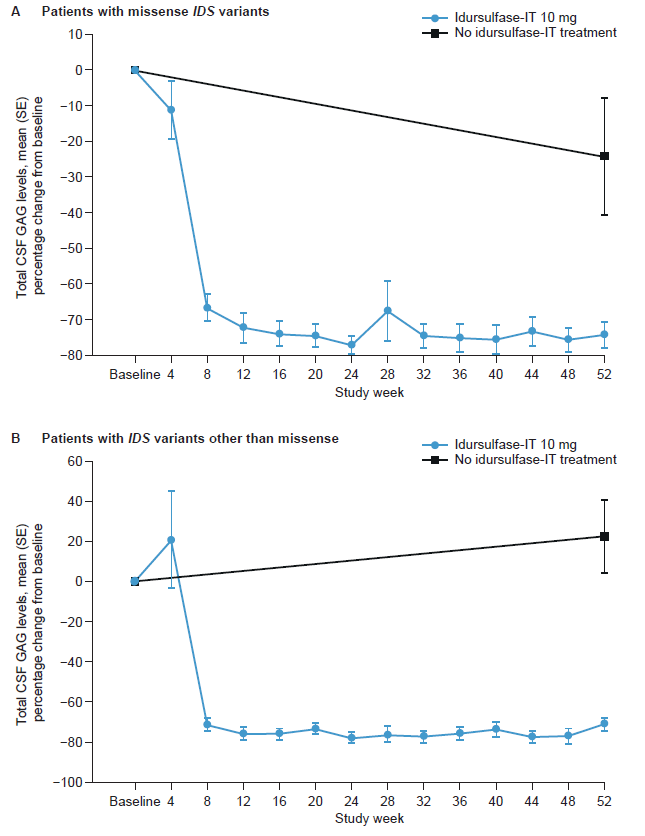


**Supplementary Fig. 4.** Mean percentage change from baseline in total CSF GAG levels over 52 weeks in patients younger than 6 years at baseline with (A) missense *IDS* variants and (B) *IDS* variants other than missense**.**

CSF, cerebrospinal fluid; GAG, glycosaminoglycan; *IDS*, iduronate-2-sulfatase gene; IT, intrathecal; SE, standard error.


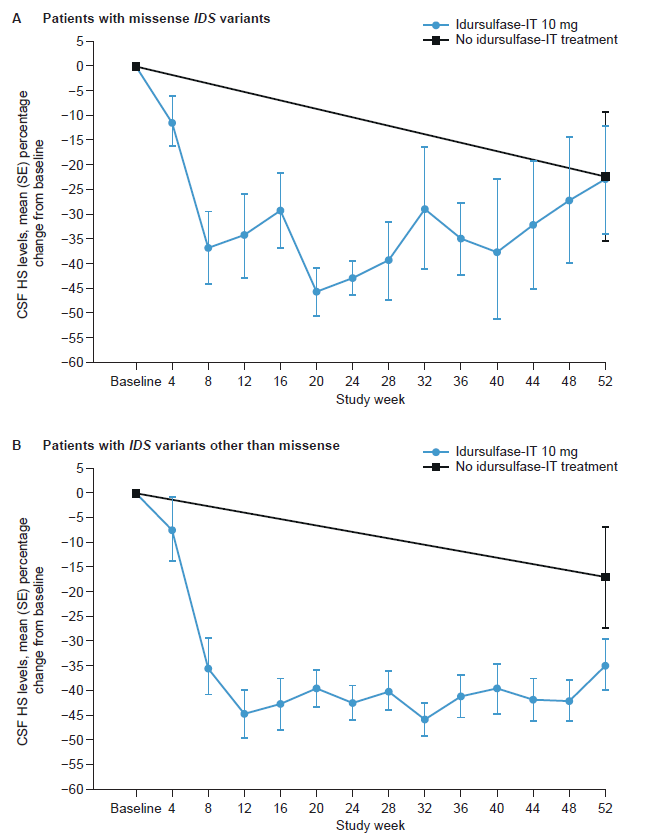


**Supplementary Fig. 5.** Mean percentage change from baseline in CSF HS levels over 52 weeks in patients younger than 6 years at baseline with (A) missense *IDS* variants and (B) *IDS* variants other than missense.

CSF, cerebrospinal fluid; HS, heparan sulfate; *IDS*, iduronate-2-sulfatase gene; IT, intrathecal; SE, standard error.


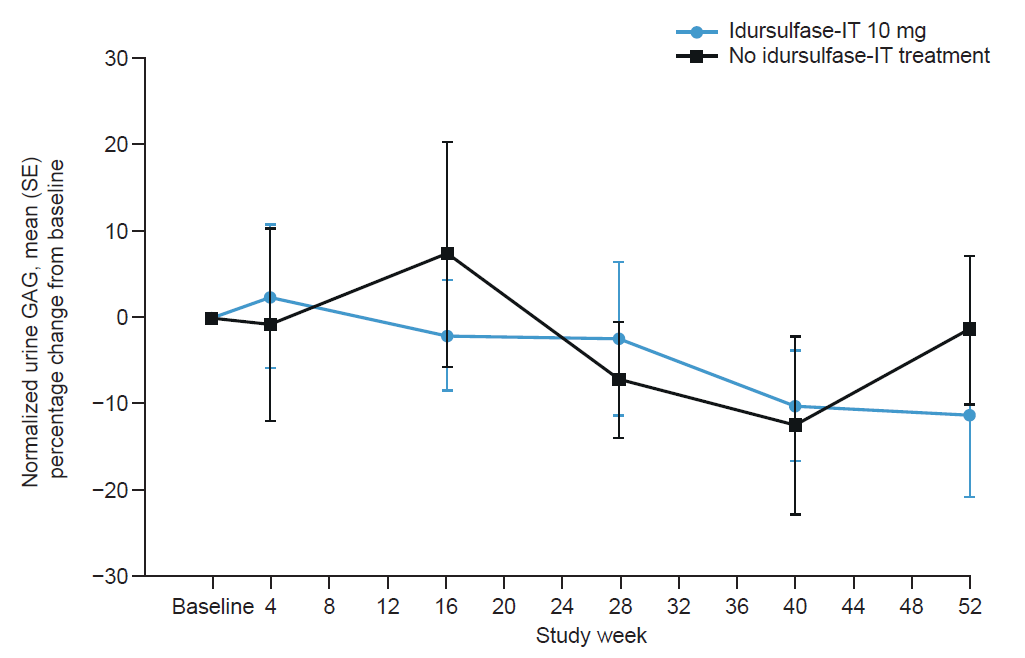


**Supplementary Fig. 6.** Mean percentage change from baseline in urine GAG levels in the ITT population over 52 weeks.

GAG, glycosaminoglycan; IT, intrathecal; ITT, intention-to-treat; SE, standard error.
